# Supplementary material for: Identification of immunodominant linear epitopes from SARS-CoV-2 patient plasma
Source: PLoS One. 2020 Sep 9;15(9):e0238089. doi: 10.1371/journal.pone.0238089 (PMC7480855; doi:10.1371/journal.pone.0238089)
Supplement: S1 File — (PDF) [file pone.0238089.s001.pdf]

Identification of immunodominant linear epitopes from SARS-CoV-2 patient plasma

Lluc Farrera-Soler[1], Jean-Pierre Daguer[1], Sofia Barluenga[1], Oscar Vadas[2], Patrick Cohen[3], Sabrina Pagano[3], Sabine Yerly[3], Laurent Kaiser[3,4], Nicolas Vuilleumier[3], Nicolas Winssinger[1]*

SUPLEMENTARY INFORMATION

1. Design of the 200-member PNA-peptide library

The Spike ectodomain protein of SARS-CoV-2 (residues 1-1213; strain Wuhan-Hu-1; GenBank: QHD43416.1)(1) was divided into 200 different 12*mer* peptides (1-100 starting in amino acid 1 and overlapping with 6 amino acids 101-200 starting at amino acid 7). All the peptides were synthesized with a unique PNA tag to allow for microarray analysis.

```
   1 mfvflvllpl vssqcvnltt rtqlppaytn sftrgvyypd kvfrssvlhs tqdlflpffs
  61 nvtwfhaihv sgtngtkrfd npvlpfndgv yfasteksni irgwifgttl dsktqslliv
 121 nnatnvvikv cefqfcndpf lgvyyhknnk swmesefrvy ssannctfey vsqpflmdle
 181 gkqgnfknlr efvfknidgy fkiyskhtpi nlvrdlpqgf saleplvdlp iginitrfqt
 241 llalhrsylt pgdsssgwta gaaayyvgyl qprtfllkyn engtitdavd caldplsetk
 301 ctlksftvek giyqtsnfrv qptesivrfp nitnlcpfge vfnatrfasv yawnrkrisn
 361 cvadysvlyn sasfstfkcy gvsptklndl cftnvyadsf virgdevrqi apgqtgkiad
 421 ynyklpddft gcviawnsnn ldskvggnyn ylyrlfrksn lkpferdist eiyqagstpc
 481 ngvegfncyf plqsygfqpt ngvgyqpyrv vvlsfellha patvcgpkks tnlvknkcvn
 541 fnfngltgtg vltesnkkfl pfqqfgrdia dttdavrdpq tleilditpc sfggvsvitp
 601 gtntsnqvav lyqdvnctev pvaihadqlt ptwrvystgs nvfqtragcl igaehvnnsy
 661 ecdipigagi casyqtqtns prrarsvasq siiaytmslg aensvaysnn siaiptnfti
 721 svtteilpvs mtktsvdctm yicgdstecs nlllqygsfc tqlnraltgi aveqdkntqe
 781 vfaqvkqiyk tppikdfggf nfsqilpdps kpskrsfied llfnkvtlad agfikqygdc
 841 lgdiaardli caqkfngltv lpplltdemi aqytsallag titsgwtfga gaalqipfam
 901 qmayrfngig vtqnvlyenq klianqfnsa igkiqdslss tasalgklqd vvnqnaqaln
 961 tlvkqlssnf gaissvlndi lsrldkveae vqidrlitgr lqslqtyvtq qliraaeira
1021 sanlaatkms ecvlgqskrv dfcgkgyhlm sfpqsaphgv vflhvtyvpa qeknfttapa
1081 ichdgkahfp regvfvsngt hwfvtqrnfy epqiittdnt fvsgncdvvi givnntvydp
1141 lqpeldsfke eldkyfknht spdvdlgdis ginasvvniq keidrlneva knlneslidl
1201 qelgkyeqyi kwpwyiwlgf iagliaivmv timlccmtsc csclkgccsc gscckfdedd
1261 sepvlkgvkl hyt
```

Synthesis of the PNA-peptide conjugate was started with the peptide on the C’ terminus followed by a PEG spacer and followed by a unique 14*mer* coding PNA (peptide sequence shown in lower capital letters to avoid confusion with PNA sequence shown in capital letter).

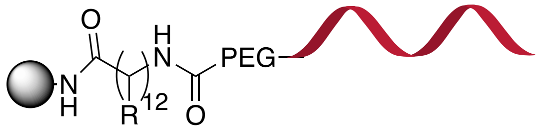

	**Peptide** N’ to C’	**PNA** C’ to N’
1	mfvflvllplvs	G C C G T G G G T G C G A A
2	sqcvnlttrtql	G C C G T G G G T G G A G A
3	yypdkvfrssvl	G C C G T G G G T G C A G G
4	hstqdlflpffs	G C C G T G G G T G G A C G
5	nvtwfhaihvsg	G C C G T G G G C A C G A A
6	tngtkrfdnpvl	G C C G T G G G C A G A G A
7	pfndgvyfaste	G C C G T G G G C A C A G G
8	ksniirgwifgt	G C C G T G G G C A G A C G
9	tldsktqslliv	G C C G T G G A C G C G A A
10	nnatnvvikvce	G C C G T G G A C G G A G A
11	fqfcndpflgvy	G C C G T G G A C G C A G G
12	yhknnkswmese	G C C G T G G A C G G A C G
13	frvyssannctf	G C C G T G G C G A C G A A
14	eyvsqpflmdle	G C C G T G G C G A G A G A
15	gkqgnfknlref	G C C G T G G C G A C A G G
16	vfknidgyfkiy	G C C G T G G C G A G A C G
17	skhtpinlvrdl	G C C G T G G A G C C G A A
18	pqgfsaleplvd	G C C G T G G A G C G A G A
19	lpiginitrfqt	G C C G T G G A G C C A G G

20	llalhrsyltpg	G C C G T G G A G C G A C G
21	dsssgwtagaaa	G C C G C C G G T G C G A A
22	yyvgylqprtfl	G C C G C C G G T G G A G A
23	lkynengtitda	G C C G C C G G T G C A G G
24	vdcaldplsetk	G C C G C C G G T G G A C G
25	ctlksftvekgi	G C C G C C G G C A C G A A
26	yqtsnfrvqpte	G C C G C C G G C A G A G A
27	sivrfpnitnlc	G C C G C C G G C A C A G G
28	pfgevfnatrfa	G C C G C C G G C A G A C G
29	svyawnrkrisn	G C C G C C G A C G C G A A
30	cvadysvlynsa	G C C G C C G A C G G A G A
31	sfstfkcygvsp	G C C G C C G A C G C A G G
32	tklndlcftnvy	G C C G C C G A C G G A C G
33	adsfvirgdevr	G C C G C C G C G A C G A A
34	qiapgqtgkiad	G C C G C C G C G A G A G A
35	ynyklpddftgc	G C C G C C G C G A C A G G
36	viawnsnnldsk	G C C G C C G C G A G A C G
37	vggnynylyrlf	G C C G C C G A G C C G A A
38	rksnlkpferdi	G C C G C C G A G C G A G A
39	steiyqagstpc	G C C G C C G A G C C A G G
40	ngvegfncyfpl	G C C G C C G A G C G A C G
41	qsygfqptngvg	G C C G G C A G T G C G A A
42	yqpyrvvvlsfe	G C C G G C A G T G G A G A
43	llhapatvcgpk	G C C G G C A G T G C A G G
44	kstnlvknkcvn	G C C G G C A G T G G A C G
45	fnfngltgtgvl	G C C G G C A G C A C G A A
46	tesnkkflpfqq	G C C G G C A G C A G A G A
47	fgrdiadttdav	G C C G G C A G C A C A G G
48	rdpqtleildit	G C C G G C A G C A G A C G
49	pcsfggvsvitp	G C C G G C A A C G C G A A

50	gtntsnqvavly	G C C G G C A A C G G A G A
51	qdvnctevpvai	G C C G G C A A C G C A G G
52	hadqltptwrvy	G C C G G C A A C G G A C G
53	stgsnvfqtrag	G C C G G C A C G A C G A A
54	cligaehvnnsy	G C C G G C A C G A G A G A
55	ecdipigagica	G C C G G C A C G A C A G G
56	syqtqtnsprra	G C C G G C A C G A G A C G
57	rsvasqsiiayt	G C C G G C A A G C C G A A
58	mslgaensvays	G C C G G C A A G C G A G A
59	nnsiaiptnfti	G C C G G C A A G C C A G G
60	svtteilpvsmt	G C C G G C A A G C G A C G
61	ktsvdctmyicg	G C C G C G A G T G C G A A
62	dstecsnlllqy	G C C G C G A G T G G A G A
63	gsfctqlnralt	G C C G C G A G T G C A G G
64	giaveqdkntqe	G C C G C G A G T G G A C G
65	vfaqvkqiyktp	G C C G C G A G C A C G A A
66	pikdfggfnfsq	G C C G C G A G C A G A G A
67	ilpdpskpskrs	G C C G C G A G C A C A G G
68	fiedllfnkvtl	G C C G C G A G C A G A C G
69	adagfikqygdc	G C C G C G A A C G C G A A
70	lgdiaardlica	G C C G C G A A C G G A G A
71	qkfngltvlppl	G C C G C G A A C G C A G G
72	ltdemiaqytsa	G C C G C G A A C G G A C G
73	llagtitsgwtf	G C C G C G A C G A C G A A
74	gagaalqipfam	G C C G C G A C G A G A G A
75	qmayrfngigvt	G C C G C G A C G A C A G G
76	qnvlyenqklia	G C C G C G A C G A G A C G
77	nqfnsaigkiqd	G C C G C G A A G C C G A A
78	slsstasalgkl	G C C G C G A A G C G A G A
79	qdvvnqnaqaln	G C C G C G A A G C C A G G

80	tlvkqlssnfga	G C C G C G A A G C G A C G
81	issvlndilsrl	G C C G G G C G T G C G A A
82	dkveaevqidrl	G C C G G G C G T G G A G A
83	itgrlqslqtyv	G C C G G G C G T G C A G G
84	tqqliraaeira	G C C G G G C G T G G A C G
85	sanlaatkmsec	G C C G G G C G C A C G A A
86	vlgqskrvdfcg	G C C G G G C G C A G A G A
87	kgyhlmsfpqsa	G C C G G G C G C A C A G G
88	phgvvflhvtyv	G C C G G G C G C A G A C G
89	paqeknfttapa	G C C G G G C A C G C G A A
90	ichdgkahfpre	G C C G G G C A C G G A G A
91	gvfvsngthwfv	G C C G G G C A C G C A G G
92	tqrnfyepqiit	G C C G G G C A C G G A C G
93	tdntfvsgncdv	G C C G G G C C G A C G A A
94	vigivnntvydp	G C C G G G C C G A G A G A
95	lqpeldsfkeel	G C C G G G C C G A C A G G
96	dkyfknhtspdv	G C C G G G C C G A G A C G
97	dlgdisginasv	G C C G G G C A G C C G A A
98	vniqkeidrlne	G C C G G G C A G C G A G A
99	vaknlneslidl	G C C G G G C A G C C A G G
100	qelgkyeqyikw	G C C G G G C A G C G A C G
101	llplvssqcvnl	G A A C T G G G T G C G A A
102	ttrtqlppaytn	G A A C T G G G T G G A G A
103	sftrgvyypdkv	G A A C T G G G T G C A G G
104	frssvlhstqdl	G A A C T G G G T G G A C G
105	flpffsnvtwfh	G A A C T G G G C A C G A A
106	aihvsgtngtkr	G A A C T G G G C A G A G A
107	fdnpvlpfndgv	G A A C T G G G C A C A G G
108	yfasteksniir	G A A C T G G G C A G A C G
109	gwifgttldskt	G A A C T G G A C G C G A A

110	qsllivnnatnv	G A A C T G G A C G G A G A
111	vikvcefqfcnd	G A A C T G G A C G C A G G
112	pflgvyyhknnk	G A A C T G G A C G G A C G
113	swmesefrvyss	G A A C T G G C G A C G A A
114	annctfeyvsqp	G A A C T G G C G A G A G A
115	flmdlegkqgnf	G A A C T G G C G A C A G G
116	knlrefvfknid	G A A C T G G C G A G A C G
117	gyfkiyskhtpi	G A A C T G G A G C C G A A
118	nlvrdlpqgfsa	G A A C T G G A G C G A G A
119	leplvdlpigin	G A A C T G G A G C C A G G
120	itrfqtllalhr	G A A C T G G A G C G A C G
121	syltpgdsssgw	G A A C C C G G T G C G A A
122	tagaaayyvgyl	G A A C C C G G T G G A G A
123	qprtfllkynen	G A A C C C G G T G C A G G
124	gtitdavdcald	G A A C C C G G T G G A C G
125	plsetkctlksf	G A A C C C G G C A C G A A
126	tvekgiyqtsnf	G A A C C C G G C A G A G A
127	rvqptesivrfp	G A A C C C G G C A C A G G
128	nitnlcpfgevf	G A A C C C G G C A G A C G
129	natrfasvyawn	G A A C C C G A C G C G A A
130	rkrisncvadys	G A A C C C G A C G G A G A
131	vlynsasfstfk	G A A C C C G A C G C A G G
132	cygvsptklndl	G A A C C C G A C G G A C G
133	cftnvyadsfvi	G A A C C C G C G A C G A A
134	rgdevrqiapgq	G A A C C C G C G A G A G A
135	tgkiadynyklp	G A A C C C G C G A C A G G
136	ddftgcviawns	G A A C C C G C G A G A C G
137	nnldskvggnyn	G A A C C C G A G C C G A A
138	ylyrlfrksnlk	G A A C C C G A G C G A G A
139	pferdisteiyq	G A A C C C G A G C C A G G

140	agstpcngvegf	G A A C C C G A G C G A C G
141	ncyfplqsygfq	G A A C G C A G T G C G A A
142	ptngvgyqpyrv	G A A C G C A G T G G A G A
143	vvlsfellhapa	G A A C G C A G T G C A G G
144	tvcgpkkstnlv	G A A C G C A G T G G A C G
145	knkcvnfnfngl	G A A C G C A G C A C G A A
146	tgtgvltesnkk	G A A C G C A G C A G A G A
147	flpfqqfgrdia	G A A C G C A G C A C A G G
148	dttdavrdpqtl	G A A C G C A G C A G A C G
149	eilditpcsfgg	G A A C G C A A C G C G A A
150	vsvitpgtntsn	G A A C G C A A C G G A G A
151	qvavlyqdvnct	G A A C G C A A C G C A G G
152	evpvaihadqlt	G A A C G C A A C G G A C G
153	ptwrvystgsnv	G A A C G C A C G A C G A A
154	fqtragcligae	G A A C G C A C G A G A G A
155	hvnnsyecdipi	G A A C G C A C G A C A G G
156	gagicasyqtqt	G A A C G C A C G A G A C G
157	nsprrarsvasq	G A A C G C A A G C C G A A
158	siiaytmslgae	G A A C G C A A G C G A G A
159	nsvaysnnsiai	G A A C G C A A G C C A G G
160	ptnftisvttei	G A A C G C A A G C G A C G
161	lpvsmtktsvdc	G A A C C G A G T G C G A A
162	tmyicgdstecs	G A A C C G A G T G G A G A
163	nlllqygsfctq	G A A C C G A G T G C A G G
164	lnraltgiaveq	G A A C C G A G T G G A C G
165	dkntqevfaqvk	G A A C C G A G C A C G A A
166	qiyktppikdfg	G A A C C G A G C A G A G A
167	gfnfsqilpdps	G A A C C G A G C A C A G G
168	kpskrsfiedll	G A A C C G A G C A G A C G
169	fnkvtladagfi	G A A C C G A A C G C G A A

170	kqygdclgdiaa	G A A C C G A A C G G A G A
171	rdlicaqkfngl	G A A C C G A A C G C A G G
172	tvlpplltdemi	G A A C C G A A C G G A C G
173	aqytsallagti	G A A C C G A C G A C G A A
174	tsgwtfgagaal	G A A C C G A C G A G A G A
175	qipfamqmayrf	G A A C C G A C G A C A G G
176	ngigvtqnvlye	G A A C C G A C G A G A C G
177	nqklianqfnsa	G A A C C G A A G C C G A A
178	igkiqdslssta	G A A C C G A A G C G A G A
179	salgklqdvvnq	G A A C C G A A G C C A G G
180	naqalntlvkql	G A A C C G A A G C G A C G
181	ssnfgaissvln	G A A C G G C G T G C G A A
182	dilsrldkveae	G A A C G G C G T G G A G A
183	vqidrlitgrlq	G A A C G G C G T G C A G G
184	slqtyvtqqlir	G A A C G G C G T G G A C G
185	aaeirasanlaa	G A A C G G C G C A C G A A
186	tkmsecvlgqsk	G A A C G G C G C A G A G A
187	rvdfcgkgyhlm	G A A C G G C G C A C A G G
188	sfpqsaphgvvf	G A A C G G C G C A G A C G
189	lhvtyvpaqekn	G A A C G G C A C G C G A A
190	fttapaichdgk	G A A C G G C A C G G A G A
191	ahfpregvfvsn	G A A C G G C A C G C A G G
192	sgncdvvigivn	G A A C G G C A C G G A C G
193	epqiittdntfv	G A A C G G C C G A C G A A
194	sgncdvvigivn	G A A C G G C C G A G A G A
195	ntvydplqpeld	G A A C G G C C G A C A G G
196	sfkeeldkyfkn	G A A C G G C C G A G A C G
197	htspdvdlgdis	G A A C G G C A G C C G A A
198	ginasvvniqke	G A A C G G C A G C G A G A
199	idrlnevaknln	G A A C G G C A G C C A G G

200	eslidlqelgky	G A A C G G C A G C G A C G

2. Synthesis of the PNA-Peptide conjugates

All reagents and solvents for the organic synthesis were purchased from commercial sources and were used without further purification. Automated solid phase synthesis was carried out on an Intavis AG Multipep RS instrument. MALDI-TOF Mass spectra were measured using a Bruker Daltonics Autoflex spectrometer operated in positive mode. HPLC purification was performed with an Agilent Technologies 1260 infinity HPLC using a ZORBAX 300SB-C18 column (9.4 x 250 mm).

2.1 General procedure for synthesis of the library and individual members

2.0 mg of NovaPEG® Rink amide resin (0.44 mmol/g, NovaBiochem) were added into 200 different wells of 96-well plates for the Automated solid phase synthesis. The resin was swollen in CH_2Cl_2 for 10 min and washed two times with DMF. Iterative cycles of amide coupling (**Procedure 1**), capping of the resin (**Procedure 3**) and deprotection of the main chain protecting group (**Procedure 2**) were done in order to synthesize the PNA-peptide sequences. Each member of the library was checked at the end of the synthesis and finally all members were mixed together and cleaved from the resin and deprotected using **Procedure 4**.

Procedure 1: Amide coupling

The Fmoc protected PNA monomer or amino acid (4.0 equiv, 0.2M in NMP) was incubated for 5 min with HATU (3.5 equiv) and a base solution [DIPEA (4.0 equiv) and 2,6-lutidine (6.0 equiv) NMP. The mixture was then added to the corresponding resin. After 20 minutes the mixture was filtered, the resin was washed with DMF, and a new premixed reaction solution was added to the resin for another 20 minutes. Finally, the resin was washed with 2 x DMF, 2 x CH_2Cl_2 and 2 x DMF.

Procedure 2: Fmoc deprotection

A solution of 20% piperidine in DMF was added to the resin and allowed to react for 5 minutes. The mixture was filtered, the resin was washed with DMF and the sequence was repeated a second time for another 5 minutes. Finally, the resin was washed with 2 x DMF and 2 xCH_2Cl_2 and 2 x DMF.

Procedure 3: Capping

The resin was treated with a capping mixture (0.92 mL of acetic anhydride and 1.3 mL of 2,6 lutidine in 18 mL of DMF; 10 mL of solution/g of resin) for 5 minutes. After flushing the solution, the resin was washed with 2 x DMF, 2 x CH_2Cl_2 and 2 x DMF.

Procedure 4: Cleavage from the resin and deprotection of the PNA/amino acid protecting groups

Resin (5.0 mg, 1.0 µmol) was treated with 125 µL of a mixture of TFA and scavengers (440 µL of TFA + 25 mg phenol + 25 µL water + 10 µL triisopropylsilane) for 2.5 hours. The resin was filtered and washed with TFA (50 µL) and the collected fractions of cleavage product were precipitated in cold ether (1.5 mL). After centrifugation, the pellet was vortexed with cold Et_2O (1.5 mL) and centrifuged again (14 000 rpm). The resulted pellet was dissolved in H_2O/CH_3CN (3/1, 1.5 mL) and lyophilized to obtain a white powder.

2.2 Characterization of the PNA-peptide library by MALDI. Expected molecular weights.

**Member**	MW	**26**	5442	**52**	5459	**78**	5131
**1**	5379	**27**	5325	**53**	5181	**79**	5286
**2**	5422	**28**	5304	**54**	5316	**80**	5237
**3**	5509	**29**	5426	**55**	5134	**81**	5333
**4**	5473	**30**	5277	**56**	5381	**82**	5458
**5**	5355	**31**	5271	**57**	5252	**83**	5398
**6**	5389	**32**	5379	**58**	5207	**84**	5389
**7**	5350	**33**	5296	**59**	5277	**85**	5180
**8**	5395	**34**	5171	**60**	5232	**86**	5321
**9**	5305	**35**	5384	**61**	5290	**87**	5336
**10**	5331	**36**	5309	**62**	5413	**88**	5356
**11**	5453	**37**	5411	**63**	5314	**89**	5247
**12**	5538	**38**	5476	**64**	5335	**90**	5422
**13**	5396	**39**	5205	**65**	5379	**91**	5338
**14**	5480	**40**	5308	**66**	5353	**92**	5498
**15**	5441	**41**	5242	**67**	5297	**93**	5244
**16**	5511	**42**	5528	**68**	5425	**94**	5316
**17**	5380	**43**	5210	**69**	5244	**95**	5436
**18**	5300	**44**	5351	**70**	5227	**96**	5439
**19**	5377	**45**	5196	**71**	5299	**97**	5133
**20**	5344	**46**	5463	**72**	5297	**98**	5483
**21**	5044	**47**	5253	**73**	5223	**99**	5317
**22**	5524	**48**	5386	**74**	5125	**100**	5574
**23**	5318	**49**	5120	**75**	5311	**101**	5312
**24**	5270	**50**	5263	**76**	5405	**102**	5429
**25**	5258	**51**	5260	**77**	5291	**103**	5474

**104**	5432	**129**	5340	**154**	5270	**179**	5252
**105**	5538	**130**	5392	**155**	5384	**180**	5293
**106**	5276	**131**	5320	**156**	5180	**181**	5207
**107**	5345	**132**	5266	**157**	5293	**182**	5439
**108**	5440	**133**	5319	**158**	5242	**183**	5440
**109**	5321	**134**	5306	**159**	5233	**184**	5478
**110**	5321	**135**	5339	**160**	5303	**185**	5138
**111**	5457	**136**	5284	**161**	5258	**186**	5313
**112**	5492	**137**	5235	**162**	5327	**187**	5404
**113**	5485	**138**	5582	**163**	5398	**188**	5269
**114**	5408	**139**	5454	**164**	5296	**189**	5379
**115**	5392	**140**	5095	**165**	5371	**190**	5281
**116**	5535	**141**	5462	**166**	5412	**191**	5356
**117**	5450	**142**	5386	**167**	5302	**192**	5186
**118**	5352	**143**	5307	**168**	5414	**193**	5358
**119**	5304	**144**	5258	**169**	5260	**194**	5210
**120**	5481	**145**	5362	**170**	5258	**195**	5400
**121**	5228	**146**	5239	**171**	5359	**196**	5545
**122**	5231	**147**	5420	**172**	5304	**197**	5236
**123**	5511	**148**	5312	**173**	5173	**198**	5292
**124**	5181	**149**	5216	**174**	5143	**199**	5395
**125**	5294	**150**	5194	**175**	5447	**200**	5404
**126**	5367	**151**	5333	**176**	5287		
**127**	5386	**152**	5273	**177**	5312		
**128**	5310	**153**	5331	**178**	5224		

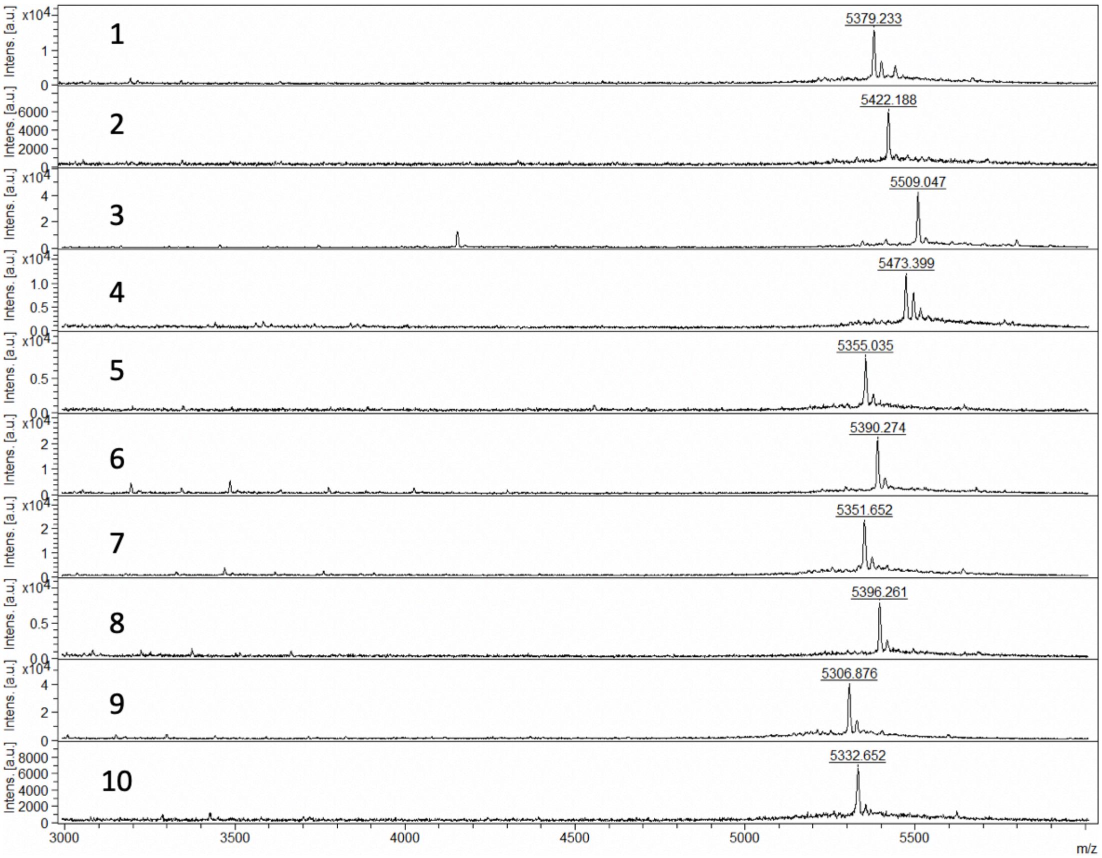
1
5379.233
2
5422.188
3
5509.047
4
5473.399
5
5355.035
6
5390.274
7
5351.652
8
5396.261
9
5306.876
10
5332.652
Intens. [a.u.]
3000
3500
4000
4500
5000
5500
m/z

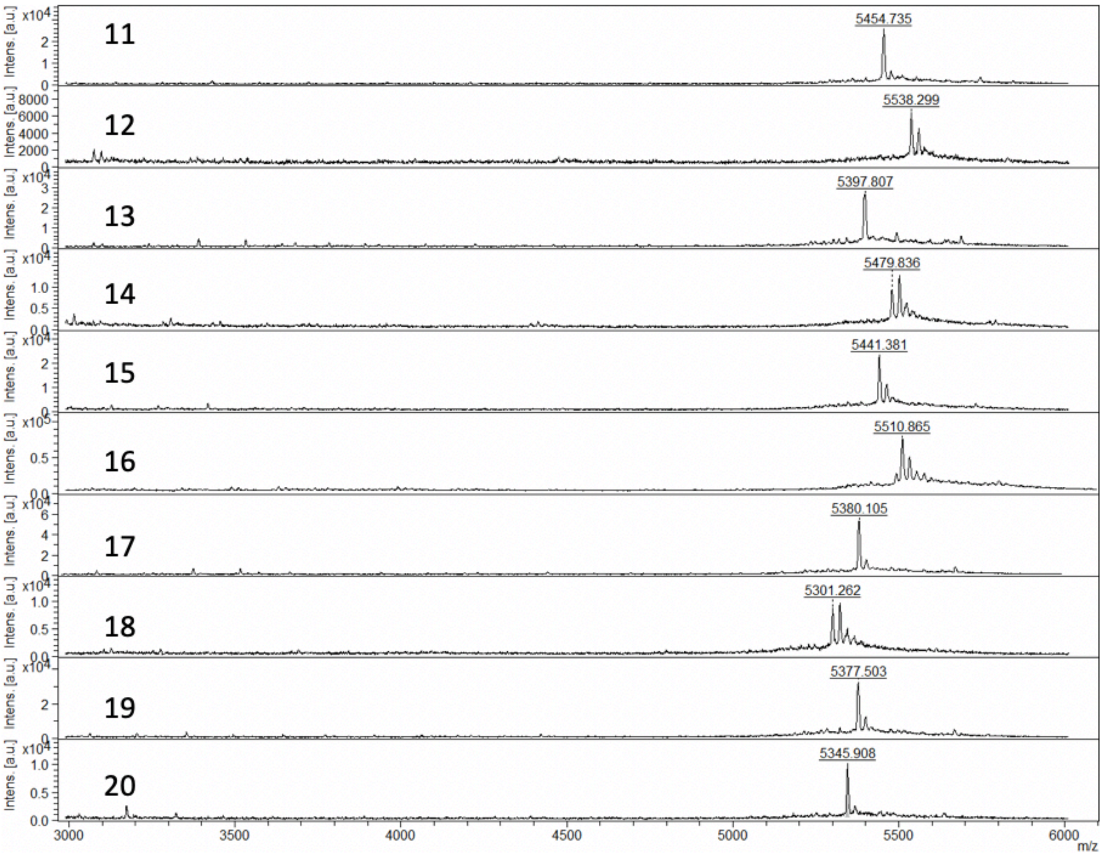
11
5454.735
12
5538.299
13
5397.807
14
5479.836
15
5441.381
16
5510.865
17
5380.105
18
5301.262
19
5377.503
20
5345.908
Intens. [a.u.]
3000
3500
4000
4500
5000
5500
6000
m/z

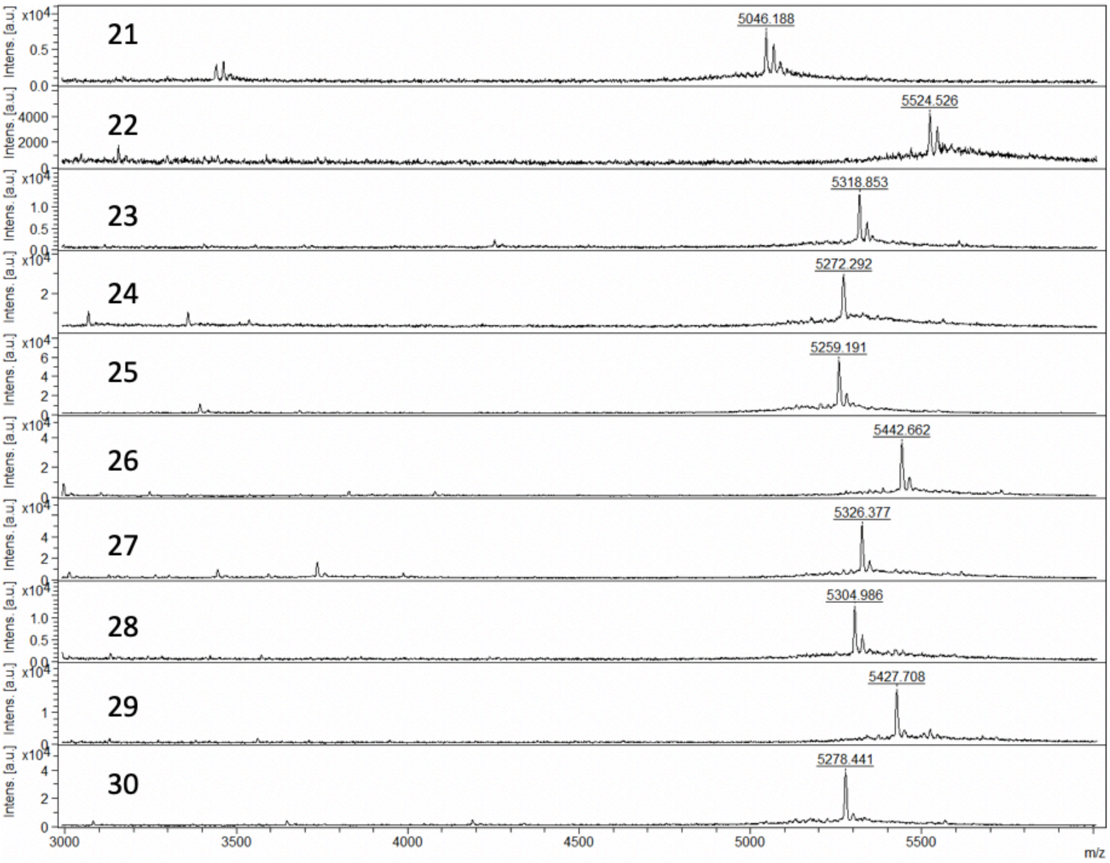

21
5046.188
22
5524.526
23
5318.853
24
5272.292
25
5259.191
26
5442.662
27
5326.377
28
5304.986
29
5427.708
30
5278.441
Intens. [a.u.]
m/z
3000
3500
4000
4500
5000
5500

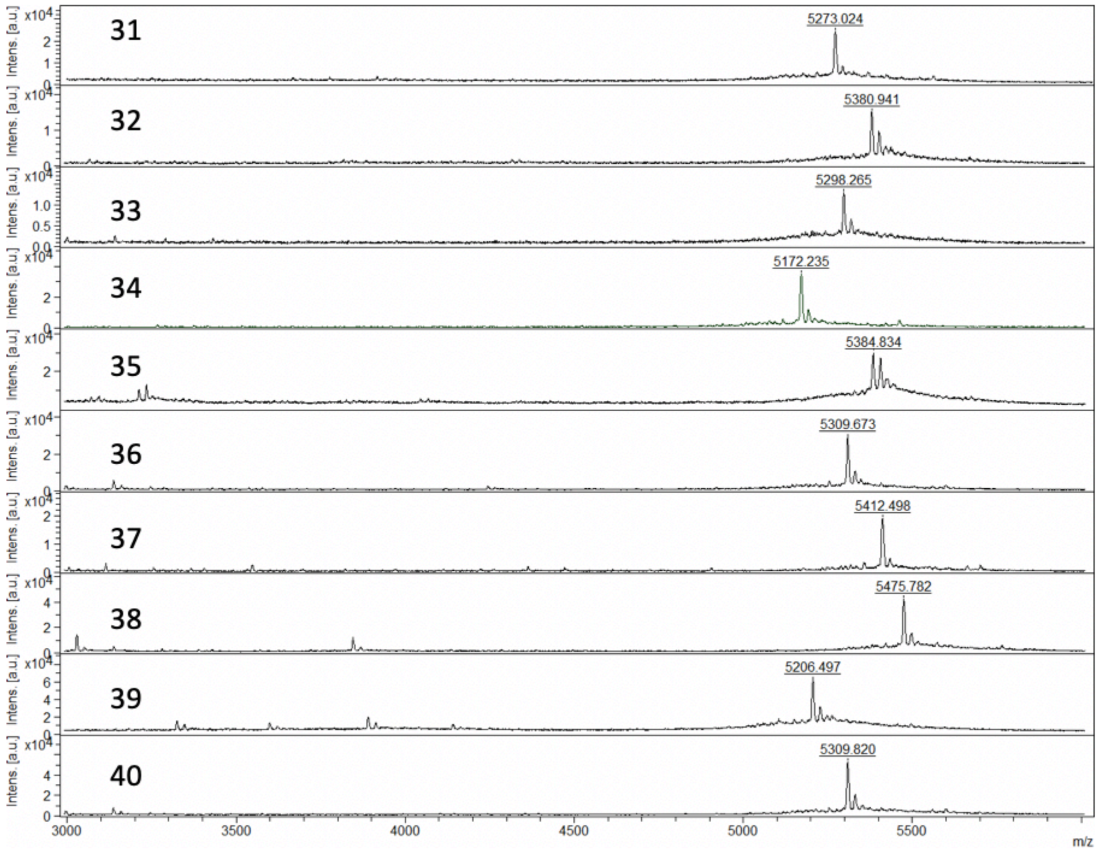

31
5273.024
32
5380.941
33
5298.265
34
5172.235
35
5384.834
36
5309.673
37
5412.498
38
5475.782
39
5206.497
40
5309.820
Intens. [a.u.]
m/z
3000
3500
4000
4500
5000
5500

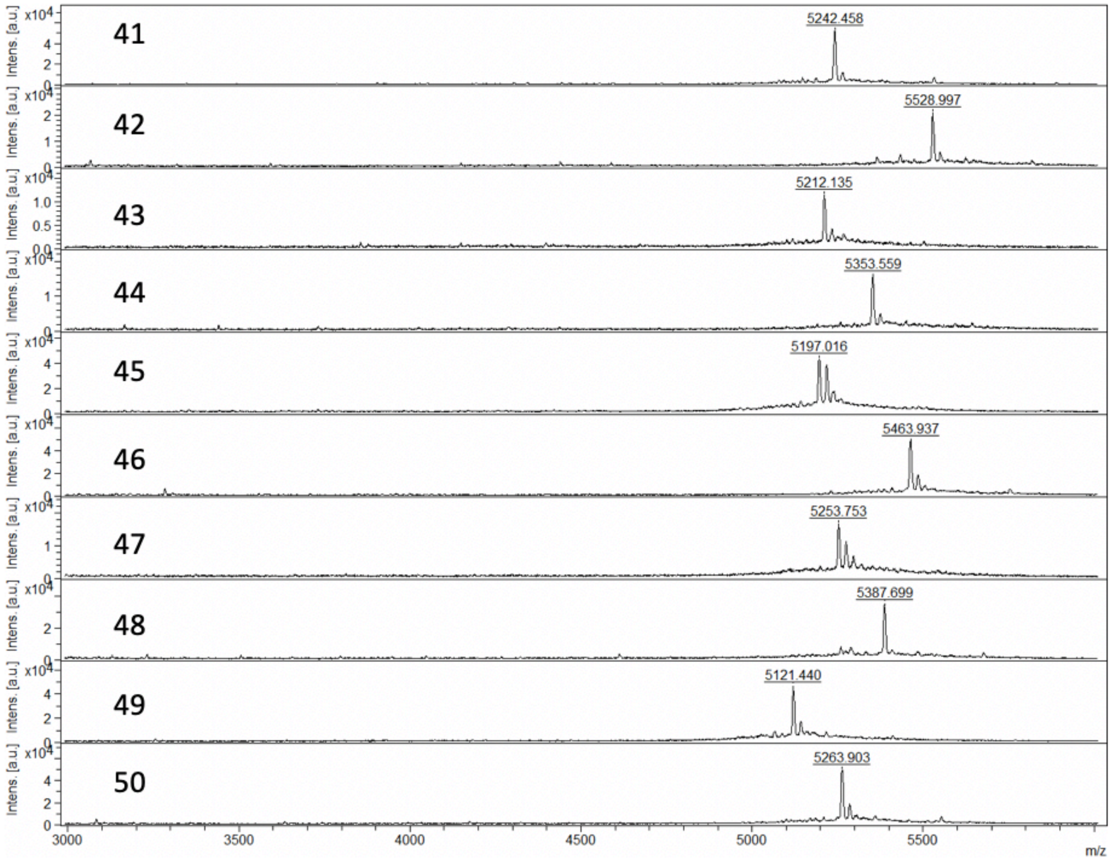
41
5242.458
42
5528.997
43
5212.135
44
5353.559
45
5197.016
46
5463.937
47
5253.753
48
5387.699
49
5121.440
50
5263.903
Intens. [a.u.]
3000
3500
4000
4500
5000
5500
m/z

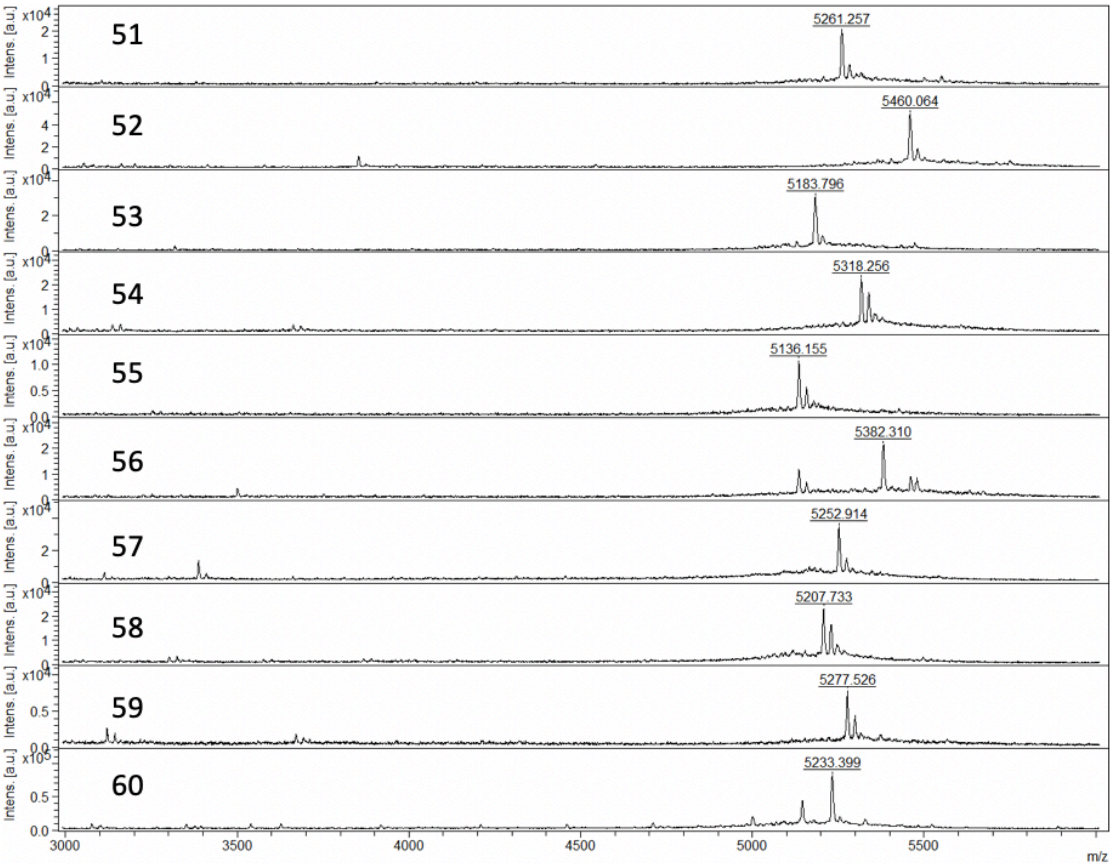
51
5261.257
52
5460.064
53
5183.796
54
5318.256
55
5136.155
56
5382.310
57
5252.914
58
5207.733
59
5277.526
60
5233.399
Intens. [a.u.]
3000
3500
4000
4500
5000
5500
m/z

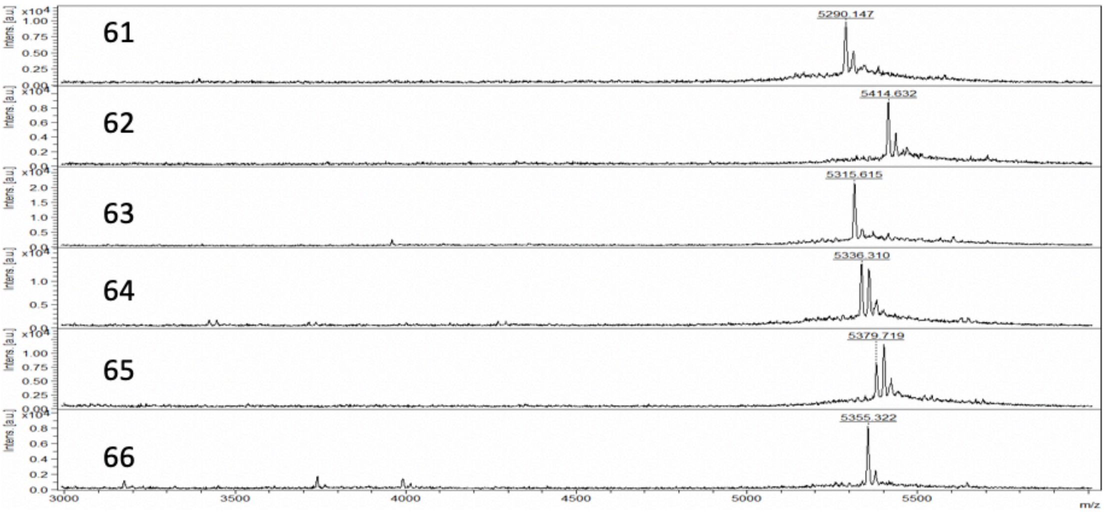
61
5290.147
62
5414.632
63
5315.615
64
5336.310
65
5379.719
66
5355.322
Intens. [a.u.]
m/z

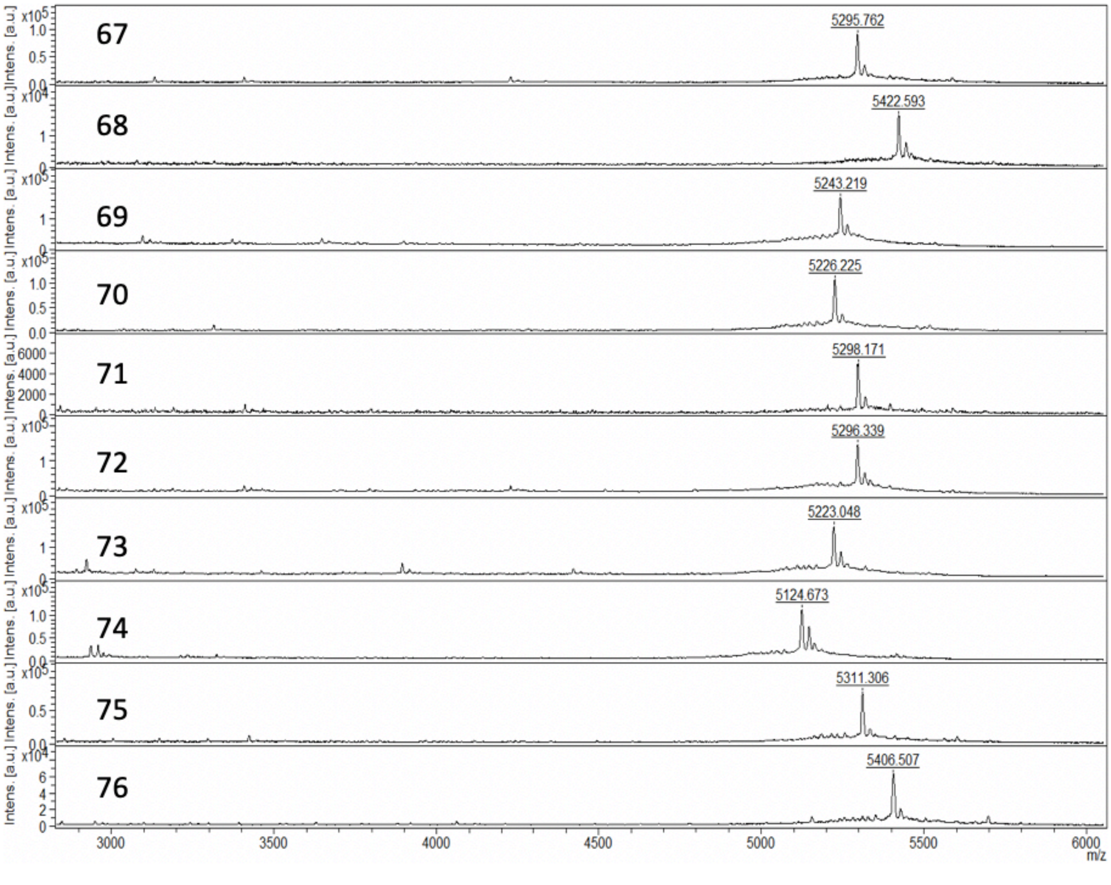
67
5295.762
68
5422.593
69
5243.219
70
5226.225
71
5298.171
72
5296.339
73
5223.048
74
5124.673
75
5311.306
76
5406.507
Intens. [a.u.]
m/z

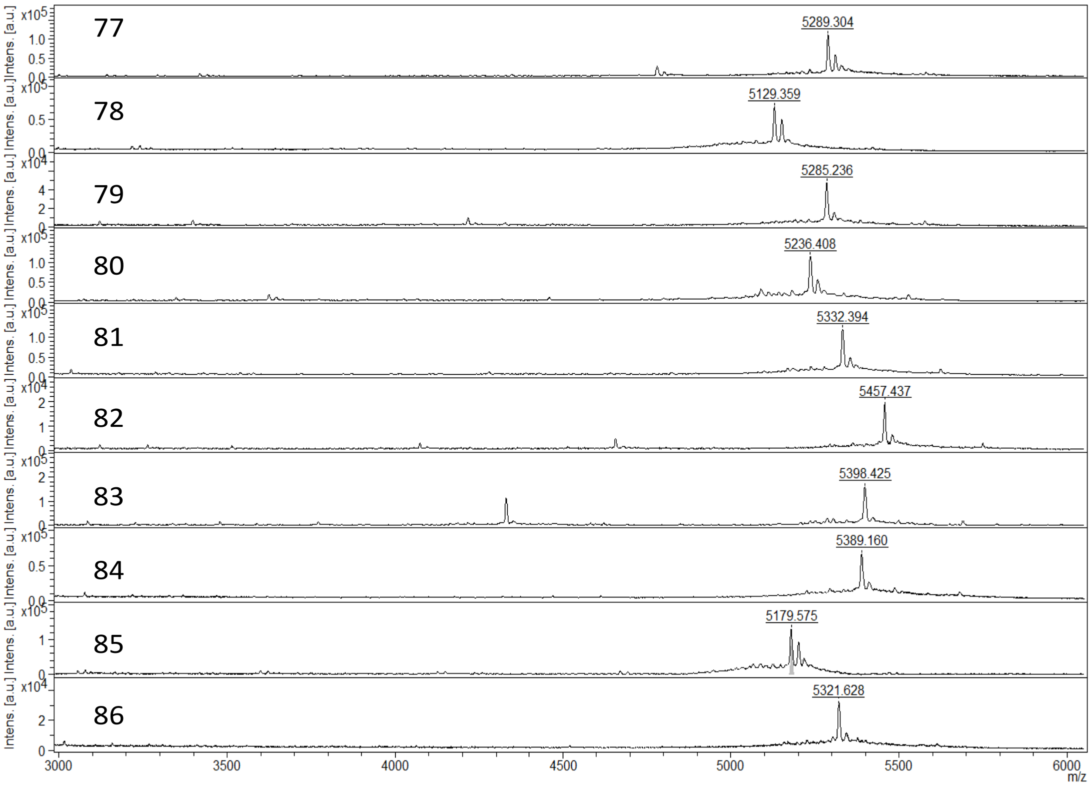

77
5289.304
78
5129.359
79
5285.236
80
5236.408
81
5332.394
82
5457.437
83
5398.425
84
5389.160
85
5179.575
86
5321.628
Intens. [a.u.]
m/z
3000
3500
4000
4500
5000
5500
6000

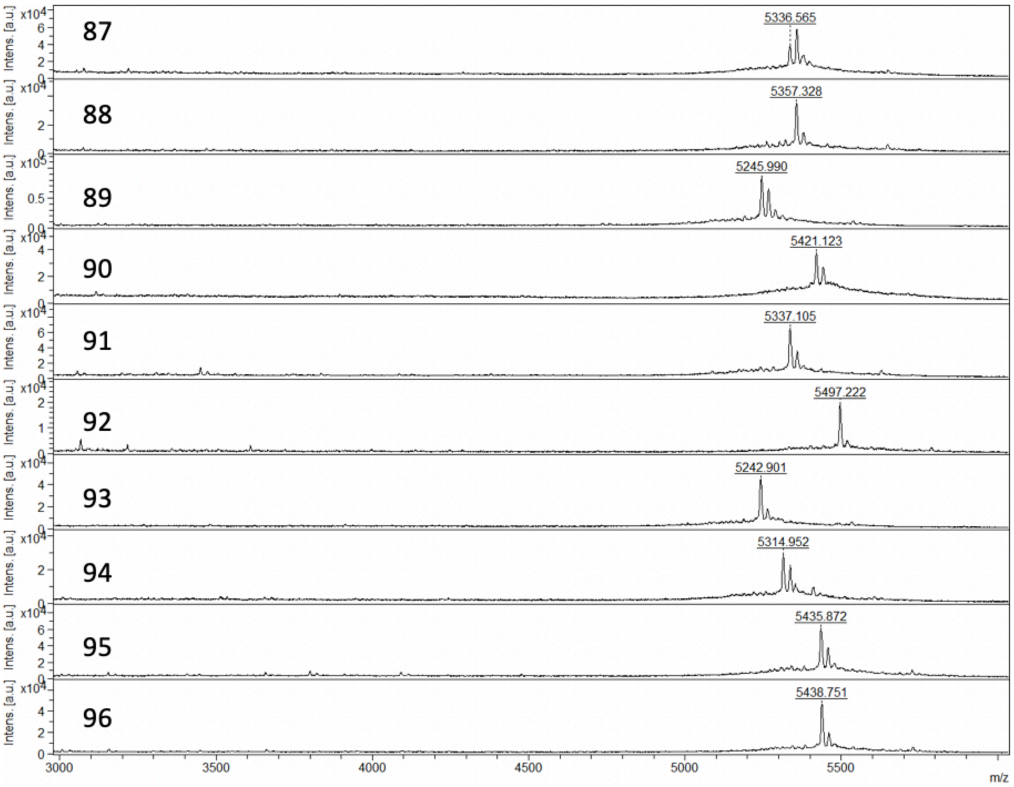

87
5336.565
88
5357.328
89
5245.990
90
5421.123
91
5337.105
92
5497.222
93
5242.901
94
5314.952
95
5435.872
96
5438.751
Intens. [a.u.]
m/z
3000
3500
4000
4500
5000
5500

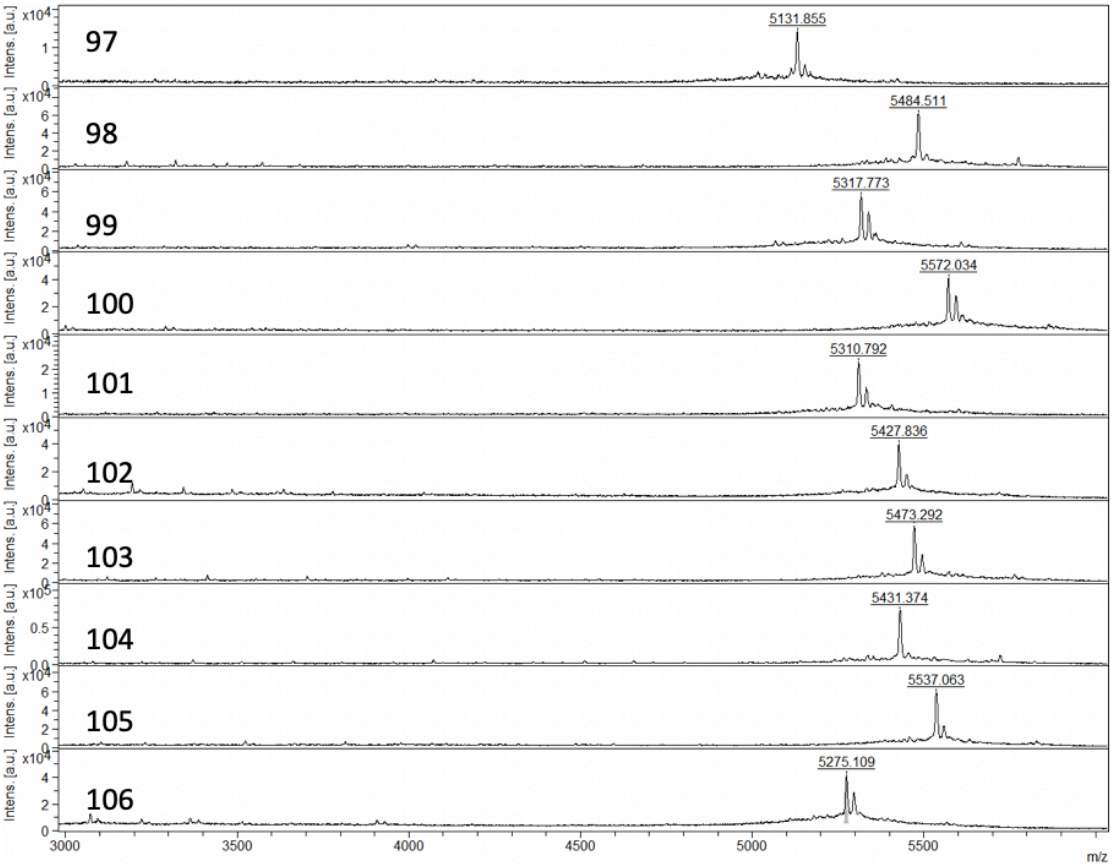
97
5131.855
98
5484.511
99
5317.773
100
5572.034
101
5310.792
102
5427.836
103
5473.292
104
5431.374
105
5537.063
106
5275.109
Intens. [a.u.]
3000
3500
4000
4500
5000
5500
m/z

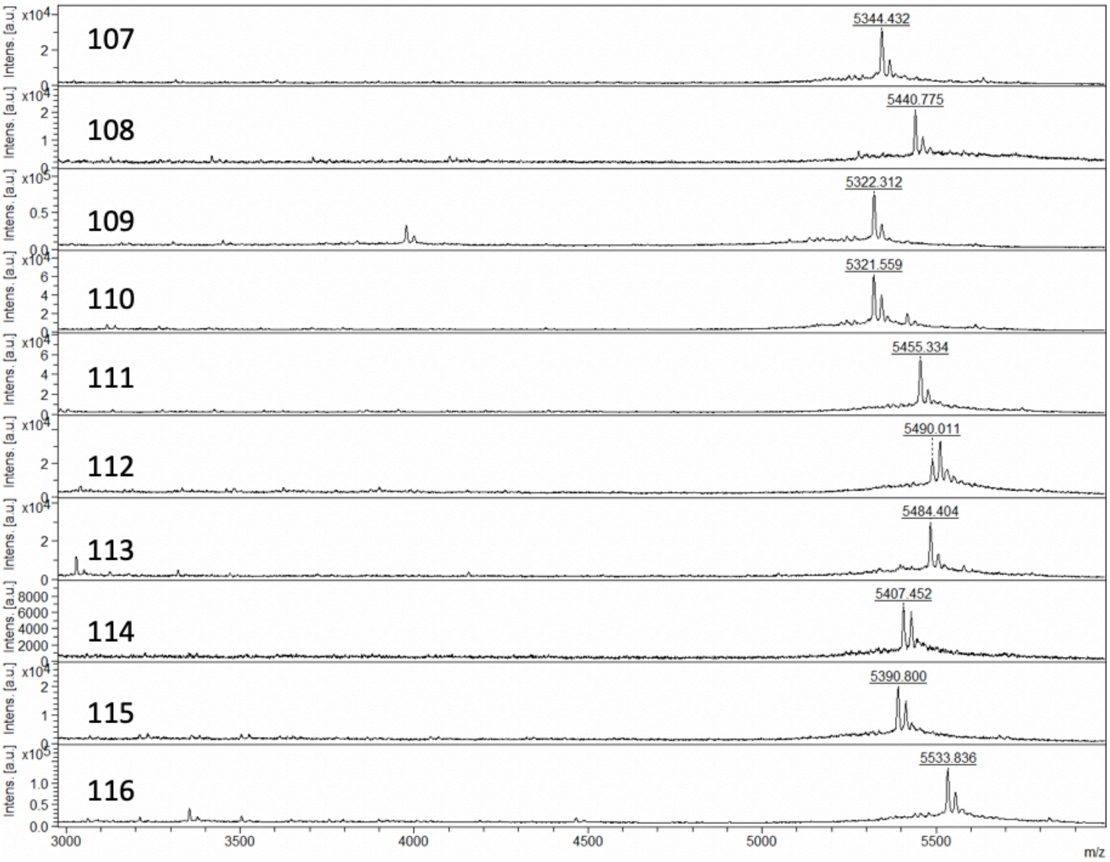
107
5344.432
108
5440.775
109
5322.312
110
5321.559
111
5455.334
112
5490.011
113
5484.404
114
5407.452
115
5390.800
116
5533.836
Intens. [a.u.]
3000
3500
4000
4500
5000
5500
m/z

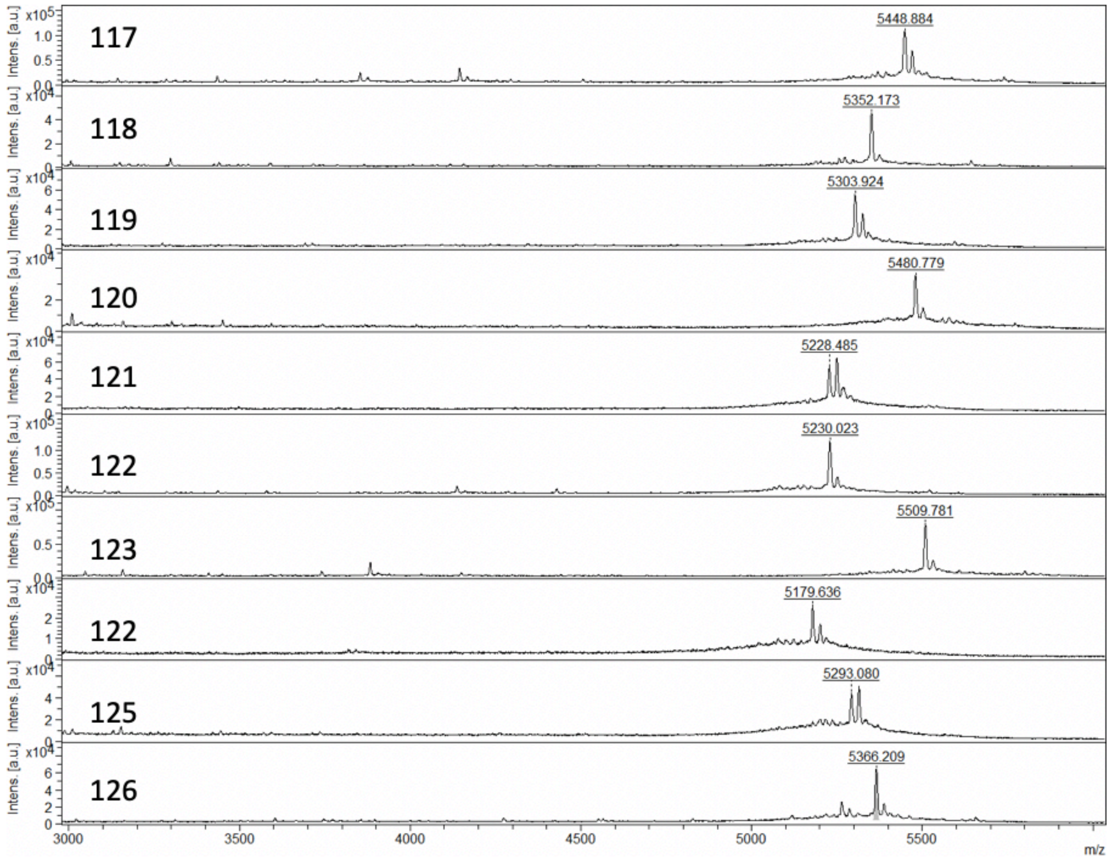
117
5448.884
118
5352.173
119
5303.924
120
5480.779
121
5228.485
122
5230.023
123
5509.781
122
5179.636
125
5293.080
126
5366.209
Intens. [a.u.]
m/z
3000
3500
4000
4500
5000
5500

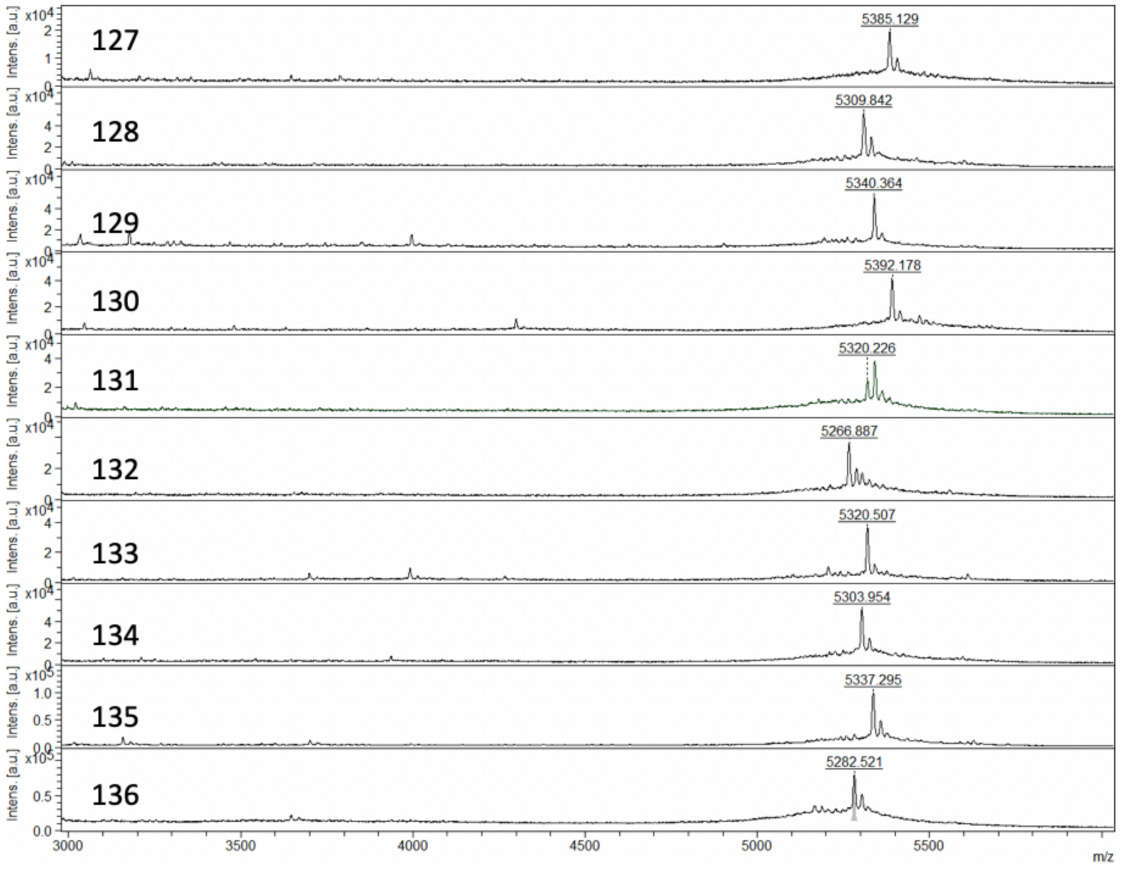
127
5385.129
128
5309.842
129
5340.364
130
5392.178
131
5320.226
132
5266.887
133
5320.507
134
5303.954
135
5337.295
136
5282.521
Intens. [a.u.]
m/z
3000
3500
4000
4500
5000
5500

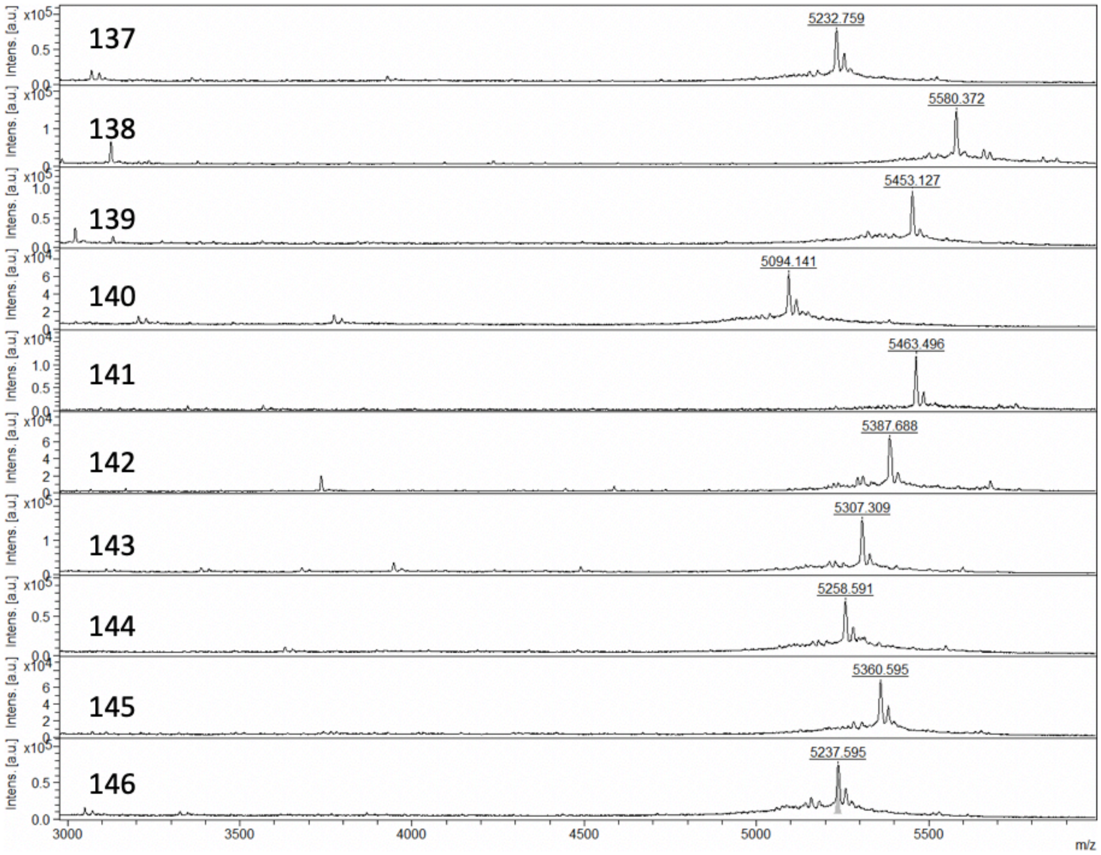

137
5232.759
138
5580.372
139
5453.127
140
5094.141
141
5463.496
142
5387.688
143
5307.309
144
5258.591
145
5360.595
146
5237.595
Intens. [a.u.]
3000
3500
4000
4500
5000
5500
m/z

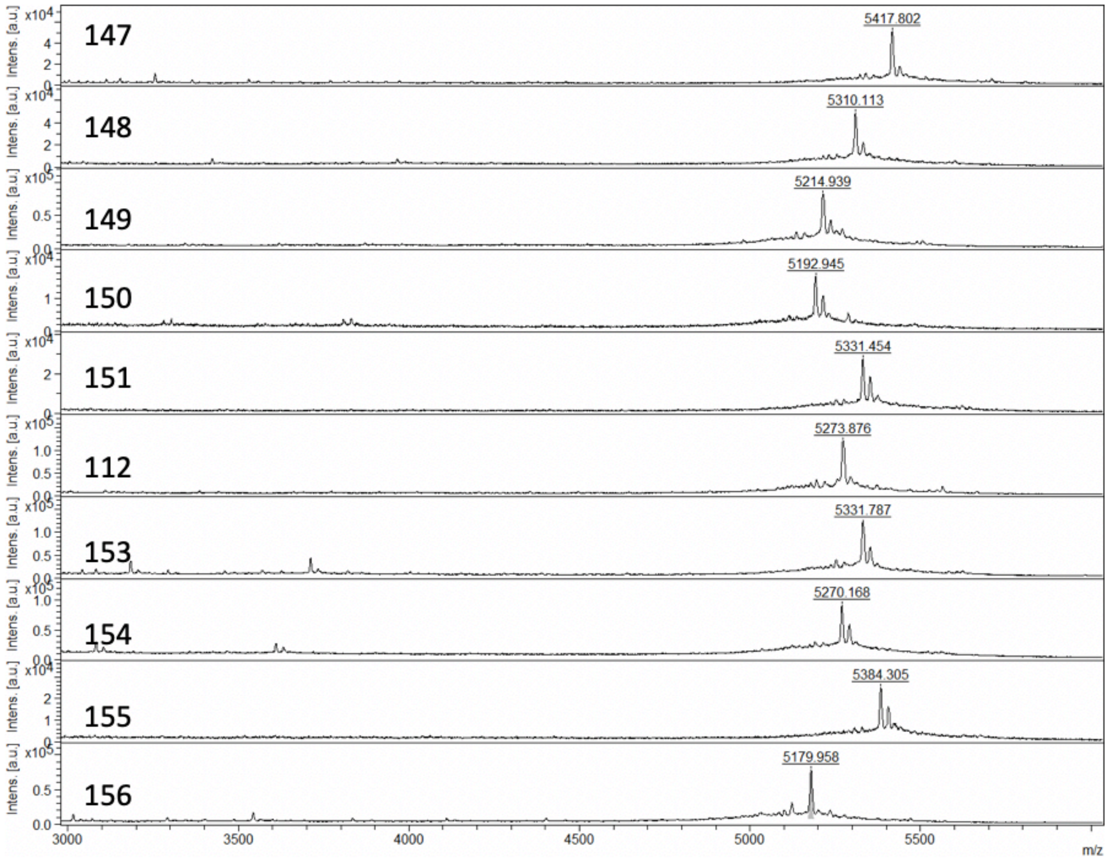

147
5417.802
148
5310.113
149
5214.939
150
5192.945
151
5331.454
112
5273.876
153
5331.787
154
5270.168
155
5384.305
156
5179.958
Intens. [a.u.]
3000
3500
4000
4500
5000
5500
m/z

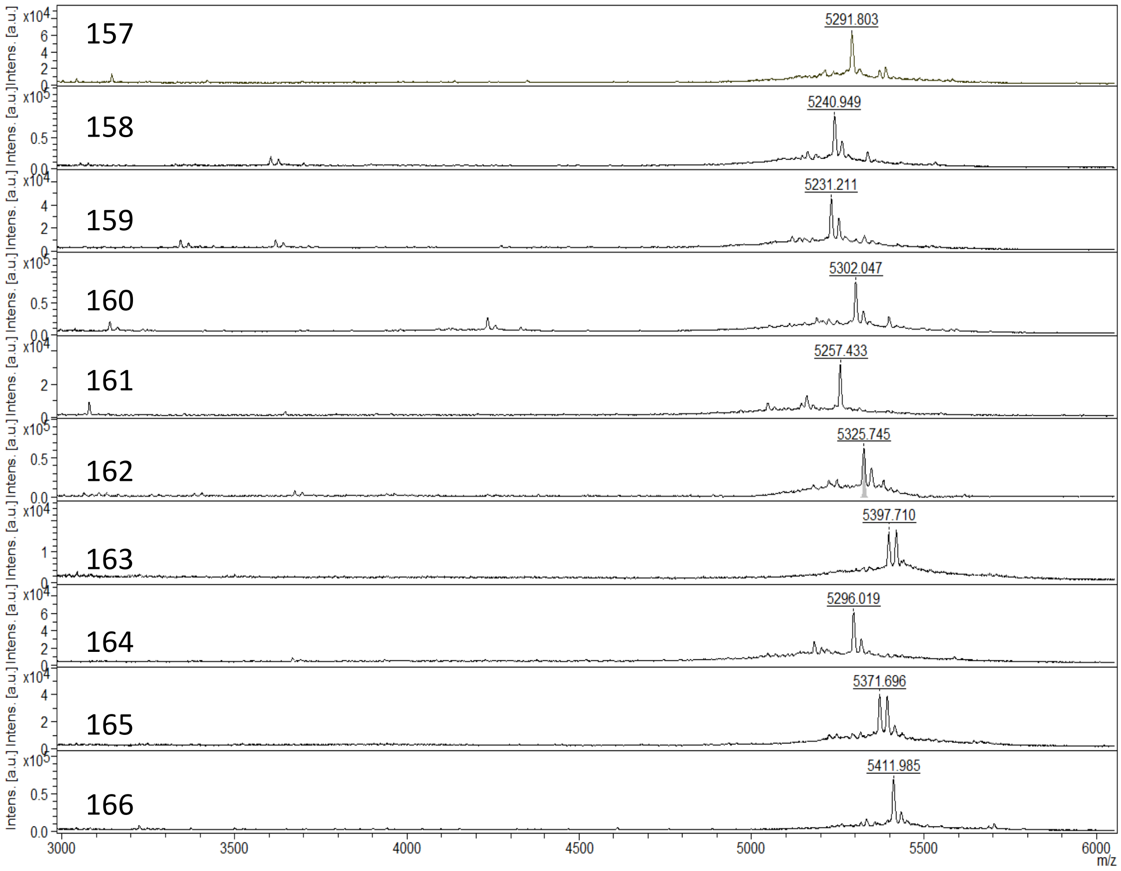
157
5291.803
158
5240.949
159
5231.211
160
5302.047
161
5257.433
162
5325.745
163
5397.710
164
5296.019
165
5371.696
166
5411.985
Intens. [a.u.]
3000
3500
4000
4500
5000
5500
6000
m/z

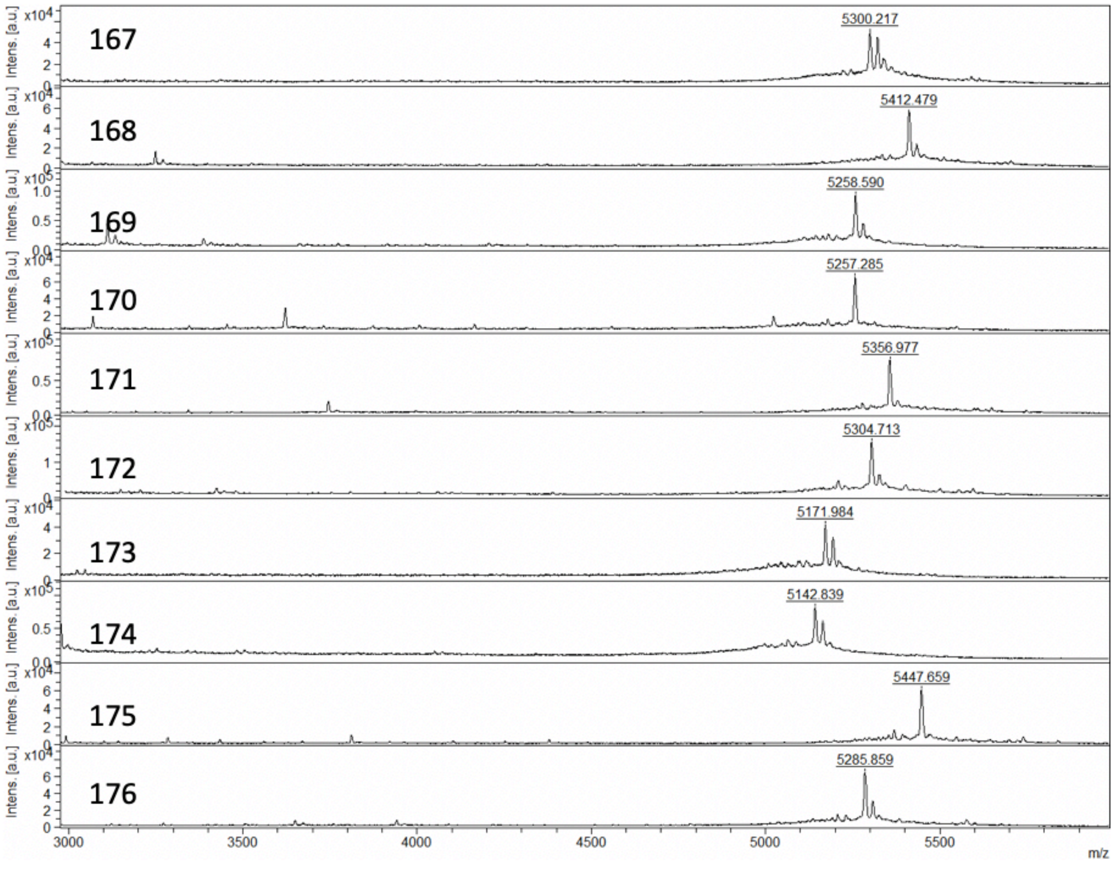
167
5300.217
168
5412.479
169
5258.590
170
5257.285
171
5356.977
172
5304.713
173
5171.984
174
5142.839
175
5447.659
176
5285.859
Intens. [a.u.]
3000
3500
4000
4500
5000
5500
m/z

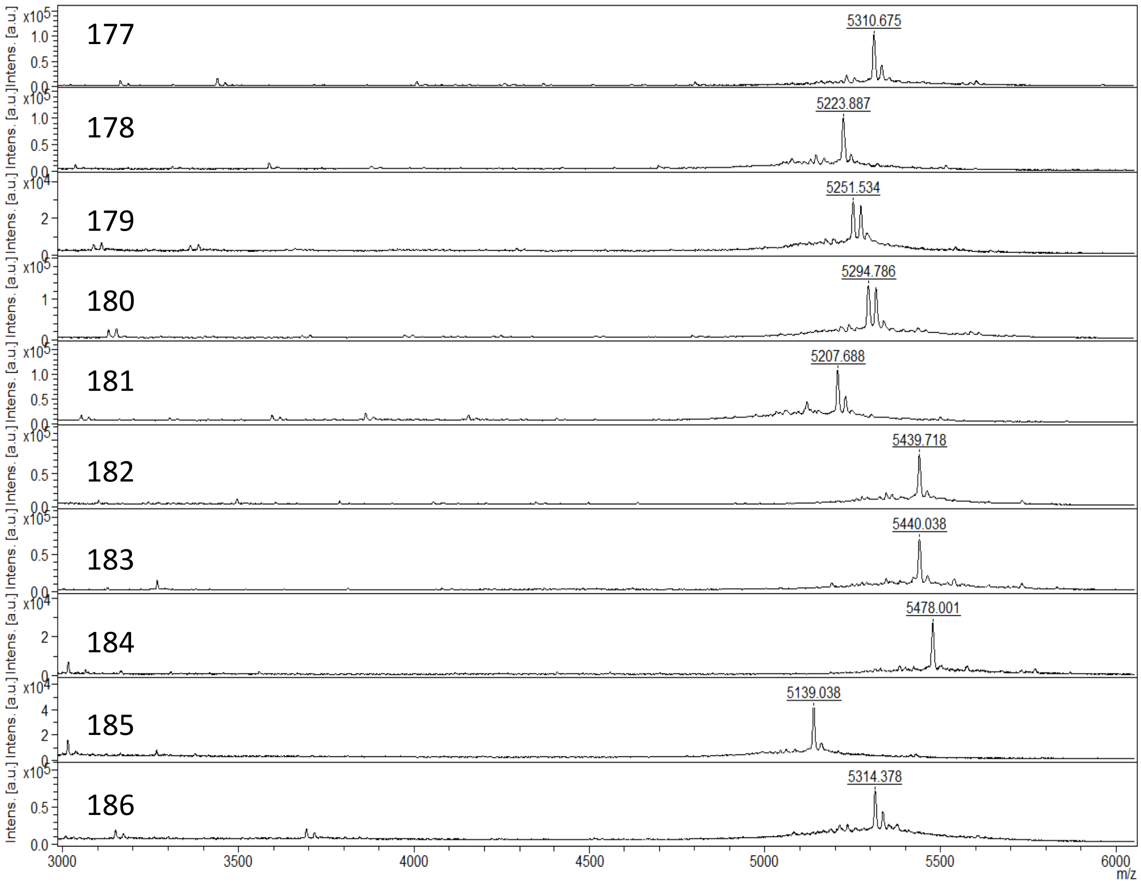

177
5310.675
178
5223.887
179
5251.534
180
5294.786
181
5207.688
182
5439.718
183
5440.038
184
5478.001
185
5139.038
186
5314.378
Intens. [a.u.]
m/z

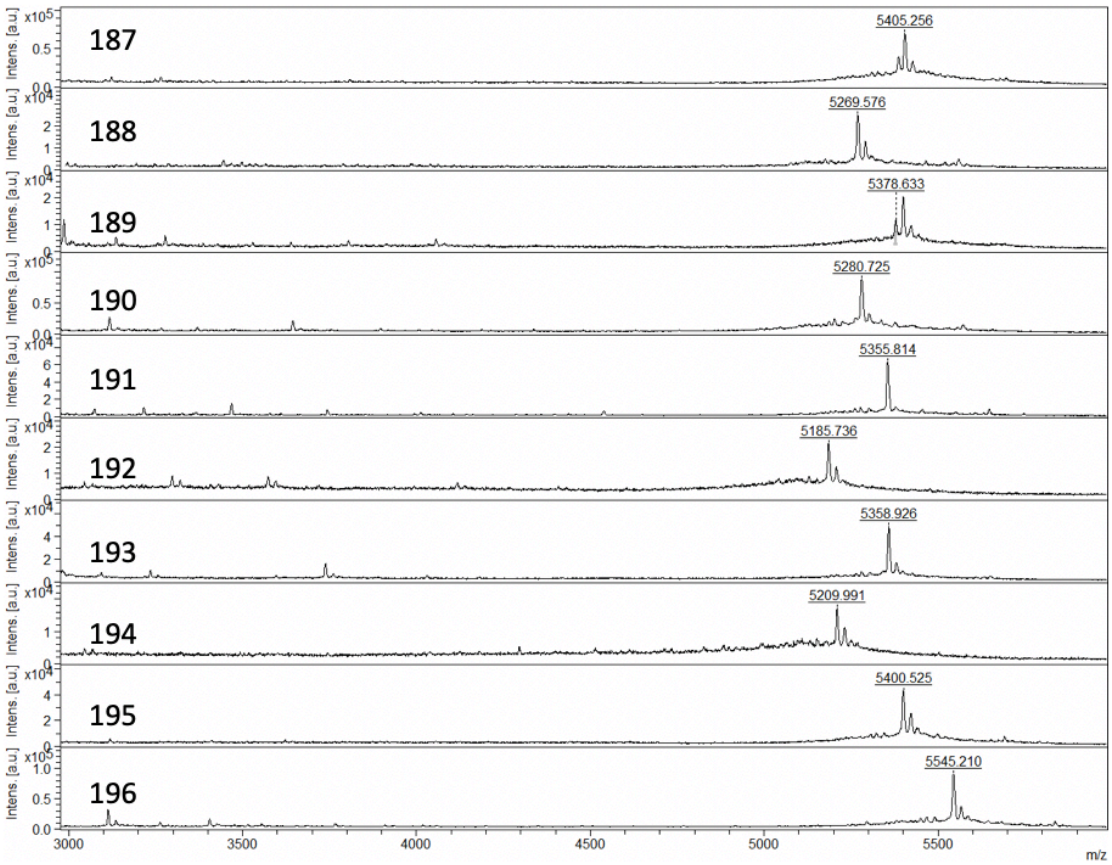

187
5405.256
188
5269.576
189
5378.633
190
5280.725
191
5355.814
192
5185.736
193
5358.926
194
5209.991
195
5400.525
196
5545.210
Intens. [a.u.]
m/z

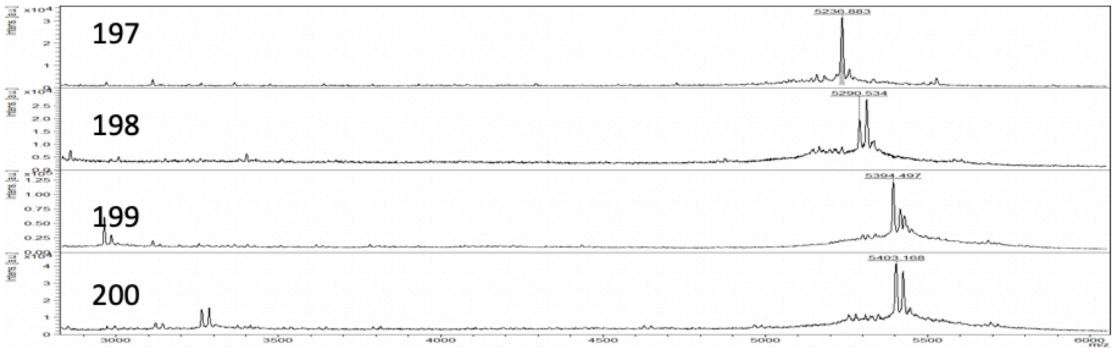

2.3 Characterization of the biotinylated peptides

Biotin-155.55: N'-Biotin-PEG-His-Val-Asn-Asn-Ser-Tyr-Glu-Cys-Asp-Ile-Pro-Ile-Gly-Ala-Gly-Ile-Cys-Ala-C'

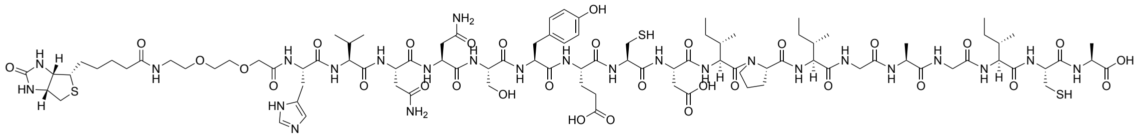

($C_{95}H_{147}N_{25}O_{32}S_3$) M^+ Isotopic peaks with relative distribution: 2247.99 (100.0%), 2246.99 (97.3%), 2248.99 (41.6%)

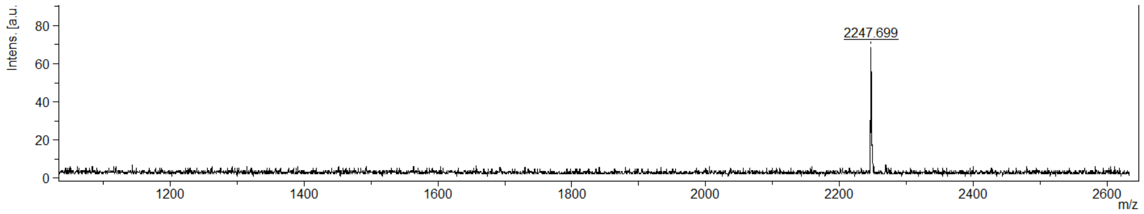

Scrambled Biotin-155.55: N'-Biotin-x-Cys-Asn-Glu-His-Ala-Ile-Tyr-Asn-Asp-Gly-Ser-Ala-Ile-Val-Cys-Ile-Gly-Pro-C'

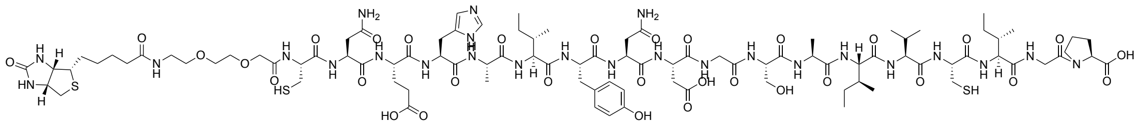

($C_{95}H_{147}N_{25}O_{32}S_3$) M^+ Isotopic peaks with relative distribution: 2247.99 (100.0%), 2246.99 (97.3%), 2249.00 (41.6%)

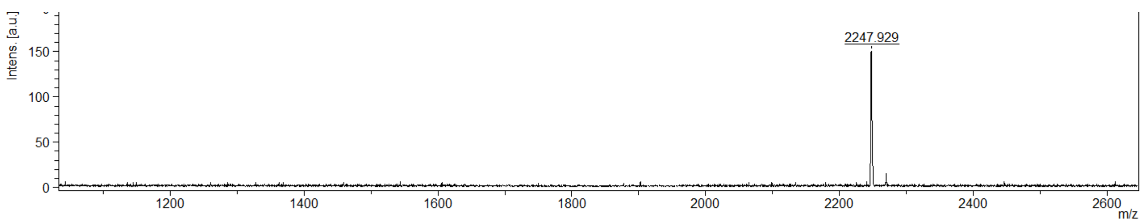

2.4 Design and characterization of the sequences for 155 Ala Scan

Ala-1: N'- AAGCGTGGGTCGGC - PEG - hvnnsyecdipigagica - C' MW=5895

Ala-2: N'- AGAGACGGCCCGGC - PEG - avnnsyecdipigagica - C' MW=5782

Ala-3: N'- AGAGGCAACGCGGC - PEG - hannsyecdipigagica - C' MW=5845

Ala-4: N'- GCAGAGCAGCCGGC - PEG - hvansyecdipigagica - C' MW=5806

Ala-5: N'- CGGACGACGGCGGC - PEG - hvnasyecdipigagica - C' MW=5822

Ala-6: N'- CGGAACGGGTCGGC - PEG - hvnnayecdipigagica - C' MW=5864

Ala-7: N'- GCAGGCAGCCCGGC - PEG - hvnnsaecdipigagica -C' MW=5733

Ala-8: N'- GCAGGTGACGCGGC - PEG - hvnnsyacdipigagica - C' MW=5822

Ala-9: N'- AAGCCGAAGCCGGC - PEG - hvnnsyeadipigagica - C' MW=5801

Ala-10: N'- AGAGGTGCGGCGGC - PEG - hvnnsyecaipigagica - C' MW=5876

Ala-11: N'- CGGAGCAGGTCGGC - PEG - hvnnsyecdapigagica - C' MW=5838

Ala-12: N'- AAGCAGCGCCCGGC - PEG - hvnnsyecdiaigagica - C' MW=5783

Ala-13: N'- AGAGAGCGGTCGGC - PEG - hvnnsyecdipagagica - C' MW=5862

Ala-14: N'- AGAGCGAGGTCGGC - PEG - hvnnsyecdipiaagica - C' MW=5918

Ala-15: N'- GCAGCGAGCCCGGC - PEG - hvnnsyecdipigaaica - C' MW=5839

Ala-16: N'- AAGCGCACGGCGGC - PEG - hvnnsyecdipigagaca - C' MW=5807

Ala-17: N'- CGGAGTGAGCCGGC - PEG - hvnnsyecdipigagiaa - C' MW=5848

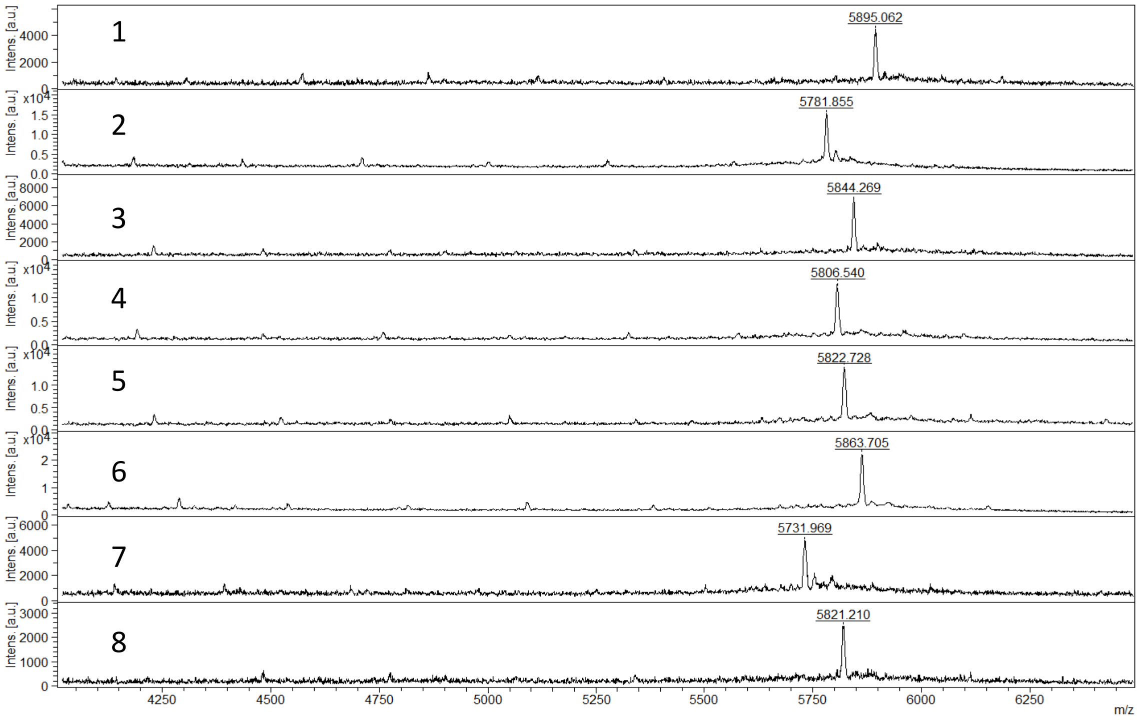

1
5895.062
2
5781.855
3
5844.269
4
5806.540
5
5822.728
6
5863.705
7
5731.969
8
5821.210
Intens. [a.u.]
4250
4500
4750
5000
5250
5500
5750
6000
6250
m/z

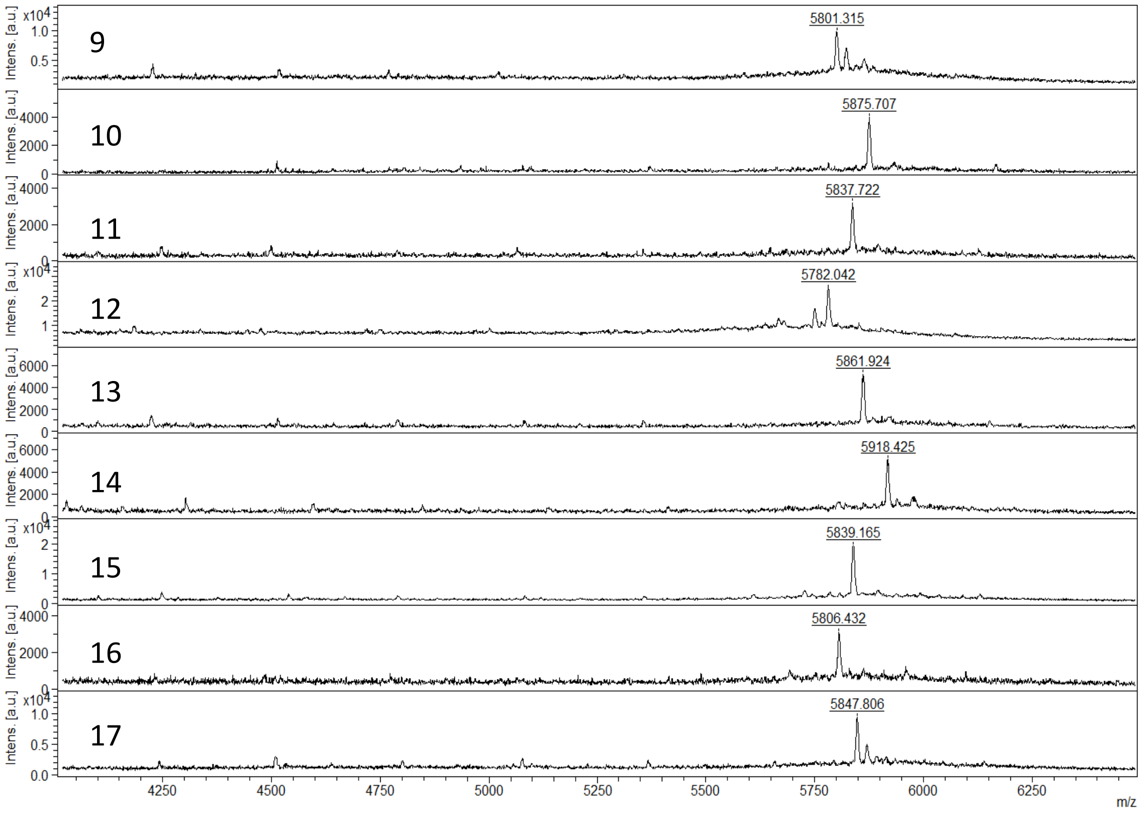

9
5801.315
10
5875.707
11
5837.722
12
5782.042
13
5861.924
14
5918.425
15
5839.165
16
5806.432
17
5847.806
Intens. [a.u.]
4250
4500
4750
5000
5250
5500
5750
6000
6250
m/z

3. Microarray screening

3.1 – Hybridization of the PNA-Peptide library

1.0 µL of a 20µM solution of PNA-peptide library in DMSO was mixed with 50 µL of hybridization buffer (1.2mM LiCl, 300mM Li-MES pH 6.1) 12mM EDTA and 3% Lithium Dodecyl Sulfate), 50 µL of Triton X-100 (Sigma-Aldrich Ref: 93443) and 1.0 µL of Salmon sperm DNA (Sigma-Aldrich Ref: D9156). This sample was heated at 95ºC for 5 minutes and centrifuged 2 minutes at 15,000g. Finally, 82 µL of the library solution were added into each array of the slide and hybridized overnight at 60ºC. After hybridization, the slides were washed for 5 minutes with SSC buffer 2x 0.1% SDS, 5 minutes with SSC buffer 0.2x 0.1%SDS and finally 30 seconds in water prior to drying by centrifugation 3 minutes at 1,000g.

3.2 – Incubation with patient's plasma samples

Analysis samples were prepared by diluting 15 µL of the plasma into 90 µL of PBS with Protease inhibitors (Promega G6521). From this diluted plasma, 5.0 µL, was further diluted into 100 µL of PBS-t-0.5%BSA and 1.0 µL of salmon sperm DNA to make a final dilution of 1:150. The mixture was centrifuged for 1 minute at 15,000g and 82 µL were added into the microarray and incubated for 1 hour at room temperature.
After incubation, the slide was washed for 5minutes with PBS-t and 30 seconds with water. Finally, the slide was dried by centrifugation for 1 minute at 1000g and ready for next step.

3.3 – Incubation of the secondary antibody
1.0 µL of Goat Anti-Human IgG H&L Cy3 (ab97170 from Abcam) was diluted with 450 µL of PBS-t and 50 µL of BSA 5% and centrifuged for 1 minute at 12krpm. This mixture, 82 µL, was added into the microarray and incubated for 30 minutes at room temperature. After incubation, the slide was washed for 5 minutes with PBS-t and 30 seconds with de

ionized water. The slide was finally dried by centrifugation for 3 minutes at 1000g and scanned on the Cy3 channel with GenePix 4100A Microarray Scanner.

3.4 – Data Analysis

Heat map of fluorescence intensity (Cy3 channel) for the screen of 200 peptide -PNA encoded library of linear epitopes from the Spike protein. The fluorescence is the median of 23 values of fluorescence quantification and then normalized to background.

4. On beads hit validation

0.3µL of Pierce™ High-Capacity Streptavidin Agarose Beads (Cat nº: 20357) were mixed with 50µL of the biotinylated peptide 10µM in PBS-t. The beads were incubated for 20' and thereafter blocked with 200µL of Fetal bovine plasma for 10'. The beads were then washed once with 100µL PBS-t and 5µL of plasma from either positive or negative patients was added together with 450µL of PBS-t and 50µL of fetal bovine plasma. The beads were incubated for 90' and after washed 4 times with 100µL of PBS-t in order to remove all the non-binders. Finally, 200µL of a 163nM solution of anti-human IgG-FITC (ref: ab6854) in PBS-t with 0.5% BSA was added and incubated for 1h. The excess of secondary antibody is washed away by washing 3 times with 100µL of PBS-t and finally the beads are imaged with Leica SP8 inverted confocal microscope (laser intensity: filters:). Quantification of FITC fluorescence was done with ImageJ by quantifying 10 different points of 10 different beads in the same image.

Images with its corresponding bright field:

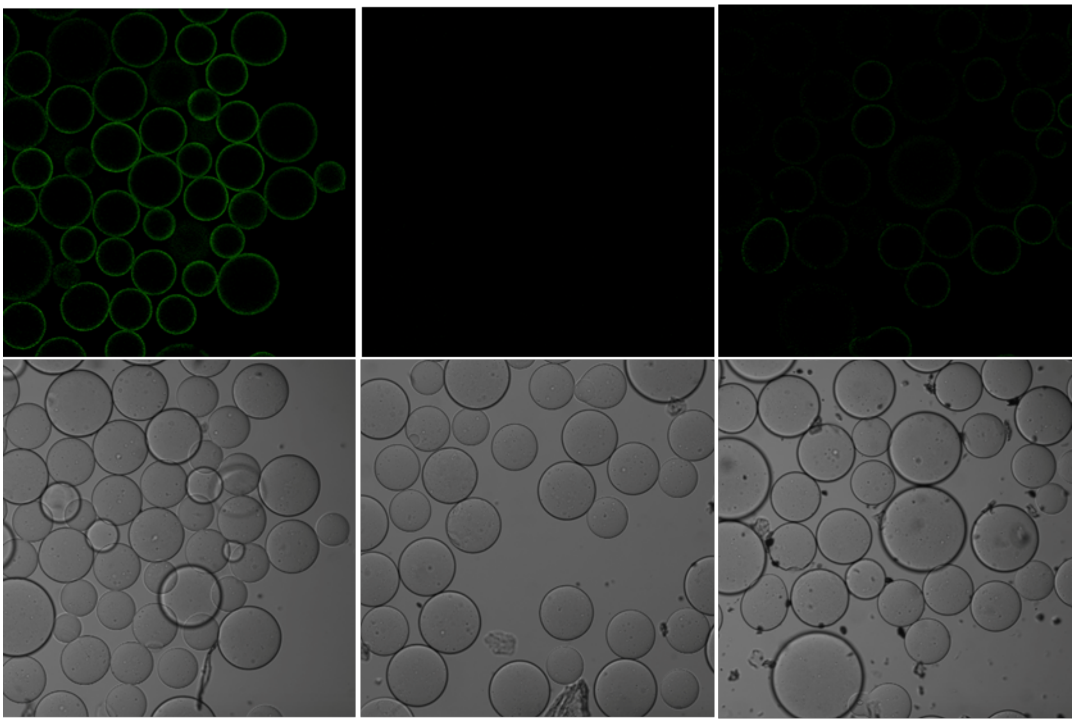

655-672 peptide (+ serum) | 655-672 peptide (- serum) | Scrambled peptide (+ serum)

5. ELISA assay

100µL of a 80nM solution of Streptavidin (Sigma Aldrich ref:S0677) in PBS were added to a Corning® 96-well Clear Flat Bottom Polystyrene High Bind Microplate (Corning, Catalog # 9018) and incubated overnight at 4ºC. The plate was then washed three times with 300µL of PBS-T (1’, rt) and 200uL of an 800nM solution of biotinylated peptide in PBS-T were added and incubated for 90’ at 36ºC. The plate was then blocked with 300µL of PBS-T with 0.5% non-fat dry milk (60’ at 36ºC). The plate was washed 3 times with 300µL of PBS-T (1’, rt) and a 1:300 diluted plasma in PBS-T-0.5% non-fat dry milk was added to each well and incubated for 90’ at 36ºC. After incubation of the plasma, the plate was washed 3 times with 300µL PBS-T (1’, rt), 1 time with PBS-T 0.5% non-fat dry milk (60’, 37ºC) and again 3 times with 300µL PBS-T (1’, rt). 100µL of Goat Anti-Human-IgG HRP conjugated (ref: ab97175) 1:10000 diluted in PBS-T 0.5% BSA were added to each well and incubated for 90’ at 37ºC. The plate was then washed 3 times with PBS-T (1’, rt) and 200µL of a 0.41mM solution of 3,3′,5,5′-Tetramethylbenzidine (TMB) (Sigma Aldrich ref: 860336) in a “50mM Na_2HPO_4, 25mM citric acid, pH 5.5 and 0.0024% H_2O_2“ solution were added to the plate and incubated for 20’ at 37 ºC. Finally, 50µL of a 1M sulfuric acid solution were added and the absorbance was measured at 450nm with a plate reader. For each sample, triplicates were done and the fluorescence value are the average of 3 reads.

6. Expended image of heat map with peptide sequence numbers

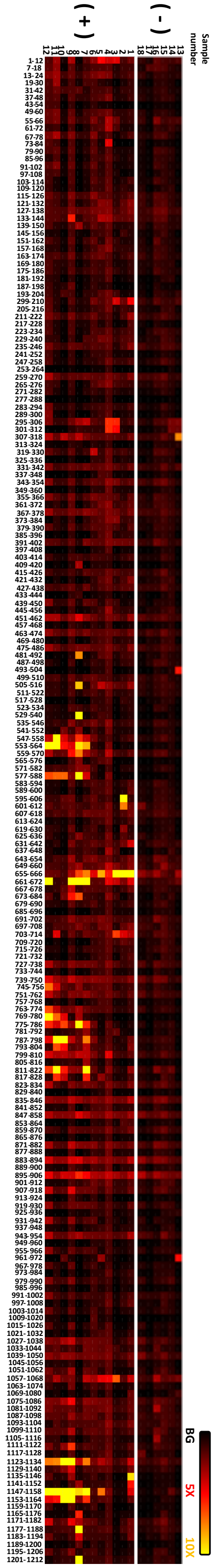

7. Spike protein furin-mediated proteolysis

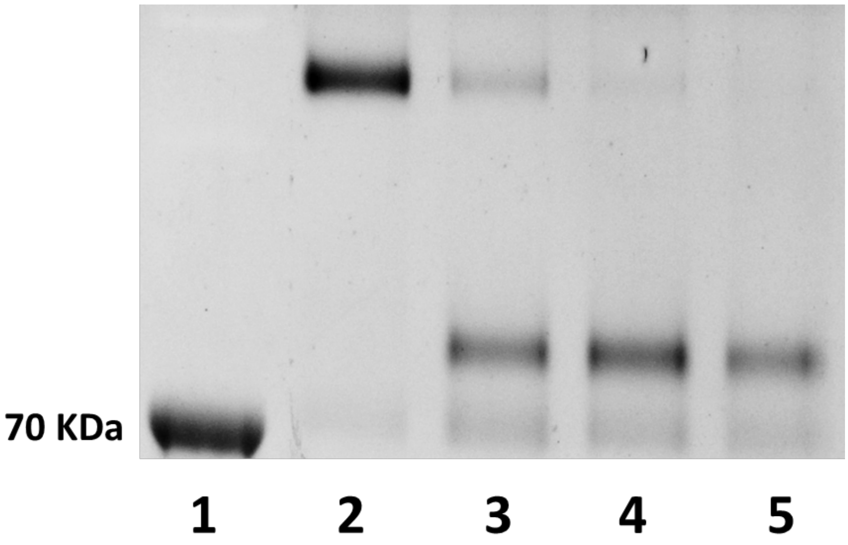

Fig S1: Fluorescence scan of a SDS-PAGE with different time points for the furin-mediated proteolysis of the spike-Dylight 459 conjugate. **Lane 1**: protein ladder, **lane 2**: Spike protein, **lane 3**: Spike protein + Furin (20'), **lane 4**: Spike protein + Furin (45'), **lane 5**: Spike protein + Furin (60').

The band corresponding to the spike protein (MW= 141'108 Da, omitting glycosylation, lane 2) is progressively converted to two new bands (expected in S1 MW=79'307 Da and S2 MW=61'819 Da, omitting glycosylation) over time (lane 3-5). The difference in fluorescence intensity of the S1 and S2 bands might correspond to different quantity of dye conjugation since the S1 domain is more exposed.

8. Different migration of the Spike protein with or without plasma

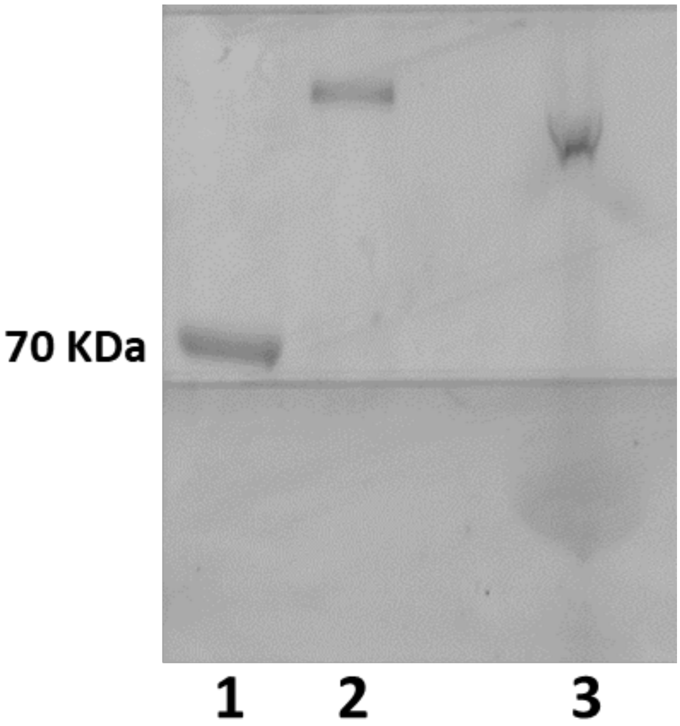

Fig S2: Fluorescent scan of a SDS-PAGE with the spike-Dylight 549 conjugate with or without plasma. **Lane 1**: protein ladder, **lane 2**: Spike protein, **lane 3**: Spike protein + plasma sample 12.

Addition of plasma (1:2 dilution) to a sample of spike-Dylight 549 conjugate results in slightly faster migration on SDS-PAGE due to the high concentration of protein loaded on the gel. Since only the spike protein is labeled with Dylight 549, the complex mixture still appears as a single band. The band corresponding to spike It can be observed the difference in migration of the Spike protein with (lane 3) and without (lane 2) plasma.

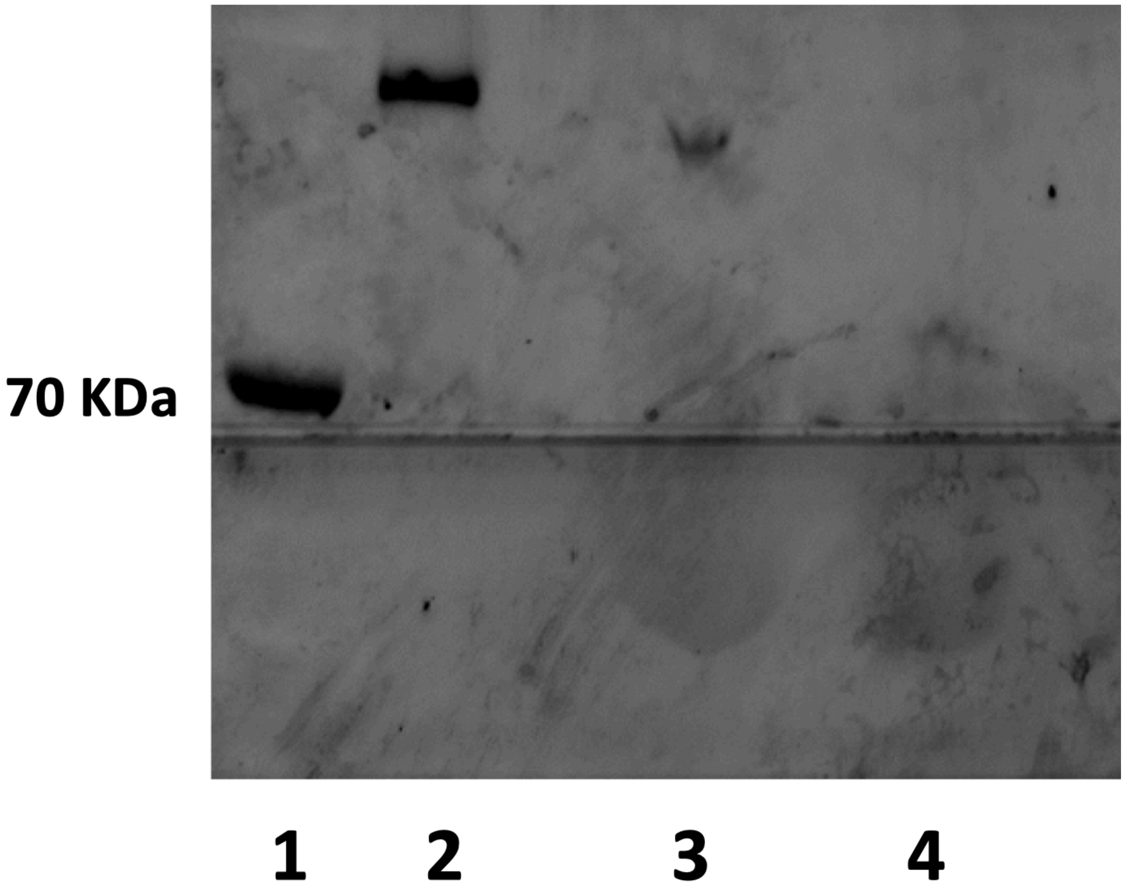

Fig S3: Inhibition of furin-mediated proteolysis of spike. Fluorescent scan of a SDS-PAGE with Dylight 549-labeled spike protein (lane 2) and treated with furin with the addition of plasma from patient 1 (positive for the epitope adjacent to the cleavage site, lane 3) and sample 14 from a healthy individual (negative for the epitope adjacent to the cleavage site, lane 4). Lane 1 is a molecule weight marker.

Supplementary Table S1

	655-672*		787-798/811-822*		1147-1158*	PCR	Serological S1	Bead 655-672	Elisa 655-672	Furin Inhibition
	HVNNSYECDIPI	ECDIPIGAGICA	QIYKTPPIKDFG	KPSKRSFIEDLL	SFKEELDKYFKN					
**1**	**30**	**5**	3	3	4	(+)	(+)			**(+)**
**2**	**12**	3	2	1	1	(+)	(+)			
**3**	**15**	4	1	2	1	(+)	(+)			
**4**	5	5	1	3	3	(+)	(+)			
**5**	**8**	2	2	2	**7**	(+)	(+)			
**6**	5	2	3	3	2	(+)	(+)			
**7**	**7**	**32**	**7**	**25**	**21**	(+)	(+)		(+)	
**8**	**6**	**36**	3	3	**9**	(+)	(+)		(+)	
**9**	4	**11**	**5**	**4**	**33**	(+)	(+)		(+)	
**10**	2	2	**10**	**5**	**28**	(+)	(+)			**(-)**
**11**	3	2	**23**	**16**	**48**	(+)	(+)			
**12**	3	**17**	**5**	**6**	**37**	(+)	(+)			**(+)**
**13**	4	3	1	1	2	(-)	(-)			
**14**	2	2	2	3	2	(-)	(-)		(-)	**(-)**
**15**	2	2	2	2	2	(-)	(-)		(-)	
**16**	3	2	2	3	2	(-)	(-)			
**17**	3	2	2	1	1	(-)	(-)		(-)	
**18**	3	2	2	1	1	(-)	(-)			

*Fluorescence Intensity over background in microarray analysis.

References

1. Wu F, Zhao S, Yu B, Chen YM, Wang W, Song ZG, et al. A new coronavirus associated with human respiratory disease in China. Nature. 2020;579(7798):265-9.
